# Neurotransmitter Systems in Alzheimer’s Disease

**DOI:** 10.3390/cimb48030334

**Published:** 2026-03-22

**Authors:** María Jesús Ramírez-Expósito, Cristina Cueto-Ureña, José Manuel Martínez-Martos

**Affiliations:** Experimental and Clinical Physiopathology Research Group CTS-1039, Department of Health Sciences, School of Health Sciences, University of Jaén, E23071 Jaén, Spain; mramirez@ujaen.es (M.J.R.-E.); ccueto@ujaen.es (C.C.-U.)

**Keywords:** Alzheimer’s disease, β-amyloid plaques, tau tangles, neurotransmitter systems, neuroinflammation, synaptic dysfunction, cholinergic system, glutamatergic system, biomarkers, precision medicine

## Abstract

Alzheimer’s disease (AD), the leading cause of global dementia, is a multifactorial process that goes beyond the accumulation of β-amyloid (Aβ) plaques and tau protein tangles, including glia cell-mediated neuroinflammation, vascular dysfunction, metabolic alterations, and synaptic loss. Its complex etiology also involves oxidative stress and mitochondrial dysfunction. Multiple neurotransmitter systems involved in the pathogenesis and the various cognitive and non-cognitive symptoms of AD are thus altered. The cholinergic system, historically the first to be associated with AD, suffers early degeneration and loss of neurons/receptors, correlating with cognitive impairment. The glutamatergic system, the main excitatory system, exhibits excitotoxicity due to increased extracellular glutamate and alterations in NMDA/AMPA receptor distribution, exacerbating neuronal damage. The GABAergic system, the main inhibitor, shows alterations in parvalbumin-positive interneurons, leading to hyperexcitability and dysfunction of neuronal networks. Monoaminergic systems (serotonergic, dopaminergic and noradrenergic) undergo early degeneration in key nuclei such as the raphe and locus coeruleus, contributing to the apathy, depression and sleep disturbances characteristic of AD. Other less explored systems, such as histaminergic and purinergic, are also crucial in cognitive modulation and neuroinflammation. The endocannabinoid system acts as a master modulator with neuroprotective and anti-inflammatory effects. These systems do not operate in isolation; their complex interactions generate pathological circuits that amplify neuronal dysfunction. The limited efficacy of current therapies, which are primarily symptomatic, highlights the need for multimodal approaches that may transform AD treatment toward personalized and more effective interventions.

## 1. Introduction

Alzheimer’s disease (AD) is the most common cause of dementia worldwide, accounting for 60–70% of cases. Globally, dementia affects approximately 50 to 55 million individuals, with projections estimating an increase to over 152 million by 2050 due to population aging. The global burden of this syndrome is substantial: in 2021, dementia (predominantly driven by AD) accounted for an estimated 28.3 million disability-adjusted life years (DALYs), representing a 160% increase since 1990. This burden is disproportionately distributed, with approximately two-thirds of cases residing in low- and middle-income countries. The economic impact is similarly staggering, with the global societal costs of dementia estimated at over $1.3 trillion annually [1,2,3,4,5]. Characterized by a progressive deterioration of memory, thinking and behavior, its pathophysiology has traditionally been attributed to the accumulation of β-amyloid (Aβ) plaques and neurofibrillary tangles of hyperphosphorylated tau protein in the brain [6,7]. These neurodegenerative processes can begin decades before the manifestation of cognitive symptoms [8], implying that multiple neurobiological systems must be affected.

**Figure 1:**
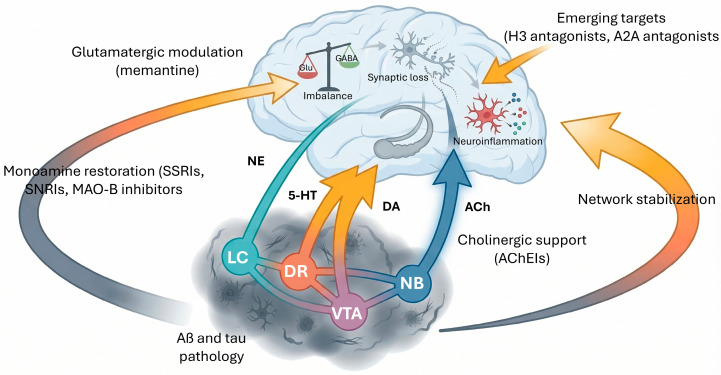
Systems-level distribution of neuromodulatory nuclei and their projections (locus coeruleus (LC; noradrenergic), dorsal raphe (DR; serotonergic), ventral tegmental area (VTA; dopaminergic), and nucleus basalis (NB; cholinergic)) converging on cortical and hippocampal circuits. AD progression reflects not a single transmitter deficit but interacting breakdowns in excitatory/inhibitory balance, synaptic integrity, and glia-mediated inflammatory signaling, which reciprocally reinforce Aβ/tau pathology and circuit dysfunction. Biomarker-guided, multimodal interventions targeting multiple nodes of neurotransmitter imbalance rather than monotherapy approaches are supportive. ACh: acetylcholine; AChEIs: acetylcholinesterase inhibitors; 5-HT: serotonin; DA: dopamine; NE: norepinephrine; Aβ: β-amyloid; Glu: glutamate; GABA: γ-amino-butyric acid; SSRIs: Selective serotonin reuptake inhibitors; SNRIs: selective noradrenaline reuptake inhibitors; MAO-B: monoamine oxidase B.

The concept of AD has evolved toward a multifactorial and systemic process, in which, in addition to the classic Aβ and tau alterations, neuroinflammatory processes, vascular dysfunction, and metabolic and synaptic alterations play an important role [9,10,11]. The importance of glia (microglia and astrocytes) and their interaction with neurotransmitter systems in disease progression is also being studied [12,13]. It has also been shown that alteration in the conventional (cholinergic, glutamatergic, GABAergic, dopaminergic, serotonergic and noradrenergic) and non-conventional neurotransmitter systems (histaminergic, purinergic and endocannabinoid) is directly involved in the accumulation of Aβ and tau, contributing to synaptic dysfunction and its clinical manifestations (Table 1). The progression of AD is increasingly understood through the interaction of these systems with soluble Aβ oligomers and specific phosphorylated tau variants, such as p-tau181 and p-tau217, which serve as early markers of network failure (Figure 1).

Specific neurotransmitter deficits in AD correlate with cognitive and non-cognitive symptoms characteristic of the disease, such as memory loss, apathy, agitation and sleep disturbances [14,15,16,17,18]. Indeed, brain areas with a high density of neurotransmitter receptors show specific atrophy linked to the progression of pathological biomarkers [19]. This fact is being exploited for the therapeutic approach to the disease [12,16,20]. Examples include the use of acetylcholinesterase inhibitors or NMDA receptor antagonists, which compensate for cholinergic and glutamatergic imbalance, respectively [21].

However, current treatments are very limited [8]. Focusing on neurotransmitter receptor alterations and acting on them to treat the disease is a promising strategy [22]. This approach has expanded in recent years with the consolidation of the use of anti-amyloid monoclonal antibodies (lecanemab, donanemab) and the development of combined strategies targeting not only the amyloid and tau pathways, but also inflammatory, metabolic and neurotransmitter homeostasis restoration processes [20,23]. Other trials combine drugs targeting both neurotransmitters (e.g., dextromethorphan with cholinergic antagonists) and inflammatory and proteostasis mechanisms, as well as a growing interest in the modulation of melatonin, nitric oxide and adenosine for their neuroprotective potential [17,24,25]. In addition, advances in circulating and imaging biomarkers have made it possible to profile patients in preclinical stages and select personalized and earlier therapies, and recent evidence indicates that modulation and restoration of balance in neurotransmitter systems will be one of the main thrusts in the treatment of AD in the coming years [20,26,27,28].

Historically, the cholinergic system was the first to be directly associated with AD. Initial investigations identified a marked loss of cholinergic neurons in the nucleus basalis of Meynert and a significant reduction in choline acetyltransferase activity in the brains of AD patients [29]. This observation gave rise to the cholinergic hypothesis, which still underpins part of the symptomatic treatment of this disease today. However, the progression of AD cannot be explained by cholinergic alterations alone, but a wide range of neurotransmitters and neuromodulators are involved, underlining the complex and multifactorial nature of the disease [20,30,31,32].

**Table 1:** Neurotransmitter system alterations and their clinical impact in Alzheimer’s Disease.

Neurotransmitter System	Key Alterations in AD	Clinical and Functional Impact	Interactions with Other Systems	References
Cholinergic	Degeneration of neurons in the Nucleus Basalis of Meynert. Reduction in ChAT activity and synaptic ACh levels. Decreased density of nAChRs (*α*7, *α*4*β*2) and mAChRs.	Impairment of attention, learning, and episodic memory. Correlates with the degree of cognitive decline.	Synergistic: ACh depletion exacerbates glutamatergic dysfunction by reducing nicotinic modulation of glutamate release. Antagonistic: α7 nAChR stimulation can reduce microglial proinflammatory cytokine release, counteracting neuroinflammation driven by purinergic P2X7 activation.	[29,33,34,35]
Glutamatergic	Excitotoxicity due to impaired astrocytic uptake (EAAT2/GLT-1 deficiency). Maladaptive redistribution of NMDA receptors (increased extrasynaptic density).	Calcium overload, mitochondrial dysfunction, and oxidative stress leading to neuronal death.	Synergistic: Excess glutamate potentiates GABAergic interneuron dysfunction through excitotoxicity. Antagonistic: Endocannabinoids (via CB1) normally suppress glutamate release; loss of CB1 in AD may disinhibit glutamate.	[6,36,37,38]
GABAergic	Vulnerability of parvalbumin-positive (PV+) interneurons. Reduced GAD expression and GABA availability. Alterations in GABAA receptor subunits (*α*2, *α*3).	Cortical hyperexcitability, loss of gamma oscillation synchronization, and increased seizure susceptibility.	Synergistic: Reduced GABAergic tone unmasks latent excitotoxicity from glutamatergic overactivity. Antagonistic: Dopamine D2 receptor activation can enhance GABA release from interneurons; loss of dopamine in VTA degeneration weakens this inhibitory control.	[39,40,41,42]
Serotonergic	Early tau-related degeneration in the Dorsal Raphe Nucleus (DRN). Reduced 5-HT levels in the hippocampus and prefrontal cortex. Reduced 5-HT1A/5-HT2A density.	Neuropsychiatric symptoms (depression, agitation, sleep disturbances) and impaired cognitive flexibility.	Synergistic: 5-HT4 receptor activation increases cAMP/PKA signaling, enhancing proteasomal degradation of tau, complementing cholinergic and dopaminergic effects on cognition.	[43,44,45,46]
Dopaminergic	Neuronal loss in the Ventral Tegmental Area (VTA). Decreased dopamine in the mesocorticolimbic pathway. Altered D1/D2 receptor density.	Apathy, executive dysfunction, and impaired reward processing. Reduced DA impairs Aβ degradation by neprilysin.	Synergistic: Dopamine promotes neprilysin-mediated Aβ degradation, a pathway that may be enhanced by noradrenergic stimulation. Antagonistic: Dopamine D1 and D2 receptors modulate GABAergic interneuron firing; loss of dopamine disinhibits hippocampal pyramidal neurons.	[47,48,49,50]
Noradrenergic	Preclinical atrophy of the Locus Coeruleus (LC). Accumulation of p-tau and the toxic metabolite DOPEGAL. Loss of anti-inflammatory control.	Deficits in arousal, attention, and stress response. Promotion of chronic neuroinflammation.	Synergistic: NE exerts anti-inflammatory effects that complement endocannabinoid-mediated neuroprotection. Antagonistic: Excess NE (paradoxically) can activate tau kinases, potentially counteracting benefits of other monoaminergic enhancers.	[51,52,53,54]
Histaminergic	Significant reduction in histamine levels. Overexpression of H3 inhibitory autoreceptors (H3R) in the hippocampus.	Sleep–wake cycle fragmentation, daytime fatigue, and cognitive slowing.	Synergistic: H3 receptor antagonism enhances release of ACh, dopamine, and NE, amplifying pro-cognitive effects across multiple systems.	[55,56,57]
Purinergic	Overexpression of A2A receptors and microglial P2X7 receptors. Elevated pathological ATP levels.	Activation of the NLRP3 inflammasome and release of IL-1β/TNF-α, exacerbating synaptic loss.	Antagonistic: A2A receptor blockade reduces glutamatergic excitotoxicity and synergizes with cholinergic enhancers. P2X7 antagonism reduces IL-1β release, complementing anti-inflammatory effects of CB2 activation.	[58,59,60,61,62]
Endocannabinoid	Decreased neuronal CB1 expression. Increased CB2 receptor density in reactive microglia.	Altered synaptic homeostasis and modulation of glial neuroinflammatory responses.	Synergistic: CB2 activation reduces microglial inflammation, complementing noradrenergic anti-inflammatory tone. CB1 activation normally suppresses glutamate release, opposing excitotoxicity.	[15,63,64,65]

## 2. Neurobiology of AD

AD is a progressive neurodegenerative condition characterized by synaptic loss, neuronal death, and brain atrophy, mainly in cortical and hippocampal regions. Its etiology is complex and involves multiple molecular and cellular processes including accumulation of misfolded proteins, neuroinflammation, oxidative stress [66], mitochondrial dysfunction, altered brain metabolism (hypometabolism), altered gene expression, synaptic dysfunction, and vascular dysregulation [2,8,67]. It affects approximately 50 million individuals worldwide, and these numbers are projected to increase to over 152 million by 2050 [2,3,5,68].

**Figure 2:**
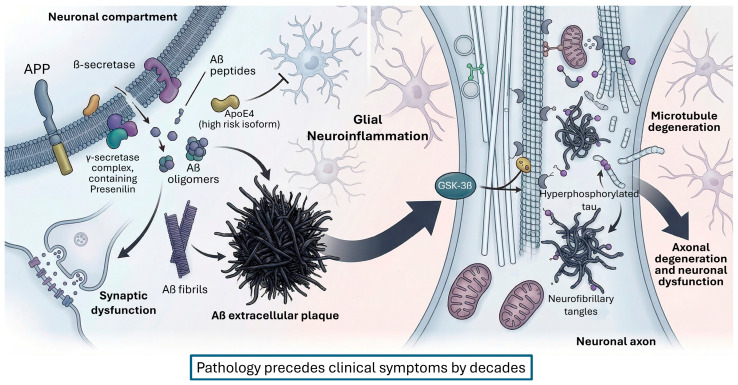
Neuropathological features of Alzheimer’s disease include extracellular Aβ plaque formation following sequential amyloid precursor protein (APP) processing by β- and γ-secretases, and intracellular aggregation of hyperphosphorylated tau into neurofibrillary tangles. Aβ oligomers/fibrils induce synaptotoxic signaling and glial activation, while tau hyperphosphorylation destabilizes microtubules and disrupts axonal transport, jointly amplifying neuroinflammation, synaptic failure, and neuronal vulnerability. Aβ/tau accumulates for years before overt cognitive symptoms, implying early circuit-level and neurotransmitter system dysfunction that precedes dementia onset.

One of the most distinctive neuropathological findings is the accumulation of extracellular Aβ plaques, derived from abnormal processing of amyloid precursor protein (APP) by β- and γ-secretases. This peptide aggregates forming insoluble oligomers and fibrils that are deposited in the extracellular space, triggering synaptic toxicity and inflammatory response [68]. In parallel, neurofibrillary tangles composed of hyperphosphorylated tau protein accumulate inside neurons, leading to destabilization of the axonal cytoskeleton and degeneration of microtubules [69,70] (Figure 2).

Recent research has evidenced that tau and amyloid affect distinct brain circuits from early stages: tau pathology, especially in the hippocampus, gives rise to memory deficits, while Aβ deposition in the amygdala is associated with early emotional disturbances such as anxiety and fear, both contributing to the clinical picture through synergistic neuroinflammatory effects and brain malfunction [71,72]. This reinforces the need to approach AD from integrated models that consider both processes as parallel and complementary drivers of pathological progression [71,73,74].

The deposition of Aβ and tau follows a stereotyped anatomical distribution. According to Braak’s criteria [52,75,76], tauopathy starts in structures such as the locus coeruleus and entorhinal cortex, and progresses to the hippocampus and neocortex. This expansion correlates closely with the clinical progression of cognitive symptoms, especially in functions such as episodic memory and spatial orientation [52,75,76,77].

Importantly, there is a time lag between the accumulation of these proteins and the appearance of clinical symptoms. This observation has led to the proposal of an amyloid cascade model, in which Aβ deposits would act as initial triggers that induce synaptic dysfunction, glial activation and secondary taupathy, ultimately causing neuronal death [76]. As a recent therapeutic strategy, selective removal of tau in brain lesions is being profiled as an adjunct to anti-amyloid immunotherapy [68]. The clinical efficacy of these therapies may depend on their ability to neutralize soluble Aβ before they trigger the GSK-3β-mediated tau phosphorylation cascade [78,79,80].

The amyloid cascade hypothesis has recently been subject to critical re-evaluation following the retraction of a highly influential study that originally identified a specific amyloid-β oligomer species (Aβ*56) as the primary memory-impairing agent. This retraction, precipitated by evidence of data and image manipulation, underscores the imperative for the rigorous validation of foundational findings and has stimulated renewed discourse regarding the precise pathological contribution of distinct Aβ assemblies. Despite this specific controversy, the broader corpus of genetic, neuropathological, and biomarker evidence continues to substantiate Aβ aggregation as a primary initiating event. Concurrently, it is increasingly recognized that downstream effector mechanisms, encompassing tau propagation, neuroinflammation, and profound neurotransmitter dysfunction, constitute the principal drivers of clinical disease progression.

Another essential component in the pathophysiology of AD is neuroinflammation. Recent evidence underlines the active role of glial cells, microglia and especially astrocytes, not only as chaperones but also as central regulators of synaptic and immune balance [73,74,81,82]. Recent findings have identified proteins such as RTP801, whose overexpression in astrocytes promotes synaptic dysfunction and cognitive impairment, and SFRP1, whose excessive accumulation in brain aging blocks the activity of the enzyme ADAM10, essential for maintaining synaptic health, and precipitates alterations in synaptic plasticity and memory consolidation [81]. These structural changes may precede and even potentiate the impact of classical amyloid plaques, acting as active drivers of pathology in silent stages [73,82]. Several genetic polymorphisms related to innate immunity such as those of TREM2, have been associated with an increased risk of AD, reinforcing the relevance of this inflammatory component [73,74,83,84,85].

Mitochondrial dysfunction and oxidative stress also play a central role in AD. Excessive reactive oxygen species (ROS) damage cellular components such as lipids, proteins and DNA, compromising neuronal viability [86,87,88,89,90]. Furthermore, reduced brain energy metabolism, evidenced by fluorodeoxyglucose (FDG) PET studies, is one of the most consistent early markers in AD [89,91,92,93].

At the synaptic level, progressive synapse loss and alterations in plasticity have been documented to be more robust predictors of cognitive impairment than Aβ or tau load alone [91]. Most novel is that dysfunction involves not only structural changes, but also a redistribution of key neurotransmitter receptors, such as NMDA receptors: recent studies in post-mortem brain tissue show a marked decrease in receptors at synapses and an increase in extrasynaptic membranes, an imbalance that favors mechanisms of toxicity and cell death, perpetuating neurodegenerative progression [36]. These alterations are largely modulated by neurotransmitter systems, whose dysfunction compromises the integrity of cortical and limbic circuits [36,94,95,96].

The cholinergic system is particularly vulnerable from the early stages of the disease. Loss of cholinergic neurons of the nucleus basalis of Meynert and decreased choline acetyltransferase activity correlate with cognitive impairment [33,34,53,97]. However, other neuronal populations are also affected, such as dopaminergic neurons in the ventral tegmental area, serotonergic neurons in the raphe, and noradrenergic neurons in the locus coeruleus, contributing to a wide range of cognitive and behavioral symptoms [46,47,50,71,98,99,100,101].

## 3. Neurotransmitter Systems in AD

### 3.1. Cholinergic System

The cholinergic system, constituted by neurons that use acetylcholine (ACh) as the main neurotransmitter, plays a fundamental role in attention, learning, memory and other cognitive functions [102,103]. Its relevance in the pathophysiology of AD has been widely recognized since early neuropathological investigations, giving rise to the so-called “cholinergic hypothesis” of AD [104]. It is now well known that ACh depletion triggers dysfunction in the posterior temporo-parietal cortexes in AD [35]. In animal models it has also been shown that decreased ACh in the hippocampus of 3xTg-AD mice correlates with cognitive impairment and depressive behavior [7], while Environmental Enrichment (EE) in 5XFAD mice increased ACh concentrations, which were found to be decreased in AD brain [105]. The study of the cholinergic system enabled the development of the first generation of drugs for this disease [1].

**Figure 3:**
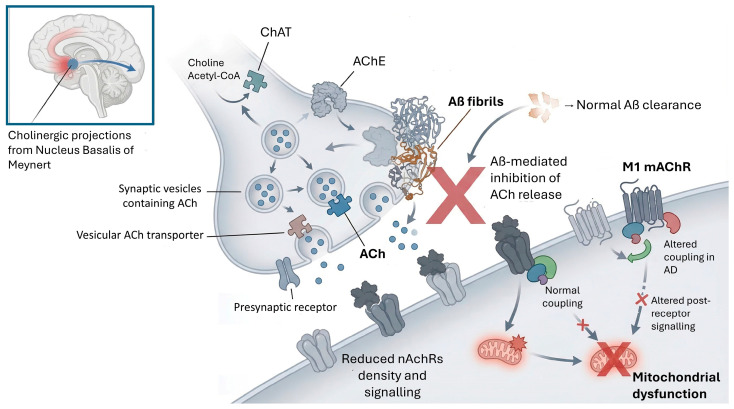
Basal forebrain cholinergic neuron degeneration, notably in the nucleus basalis of Meynert, is represented with reduced choline acetyltransferase (ChAT) activity, impaired synaptic acetylcholine (ACh) signaling, and reduced nicotinic receptor density (including α7 nAChRs). Cholinergic failure links to cognitive impairment (attention, learning, memory) and to amyloid biology, since α7 nAChR stimulation can modulate microglial cytokine release and Aβ uptake/clearance, while Aβ–receptor interactions can also mediate neurotoxic signaling.

Under normal conditions, cholinergic neurons in the basal forebrain, especially those originating in the nucleus basalis of Meynert, project to the cerebral cortex and hippocampus, modulating synaptic plasticity, neuronal excitability and information integration [106,107] (Figure 3). These neurons use choline acetyltransferase (ChAT) to synthesize ACh from choline and acetyl-CoA, and store the neurotransmitter in synaptic vesicles via the vesicular ACh transporter (VAChT). Release occurs in response to depolarization and subsequent calcium entry, followed by rapid degradation of ACh by the enzyme acetylcholinesterase (AChE) in the synaptic cleft. Acetylcholinesterase (AChE) activity is impaired in AD [108]. Treatment with AChE inhibitors such as Donepezil can induce adjustments in the cholinergic system, reducing alterations in resting-state activity secondary to ACh depletion [35].

In the brain of AD patients, a significant loss of basal forebrain cholinergic neurons, a reduction in ChAT activity, and a decrease in the density of muscarinic and nicotinic receptors have been described. These alterations correlate directly with the degree of cognitive impairment observed, especially episodic memory and attention span. Similarly, variants in genes for acetylcholine receptors are considered possible risk factors for AD [22].

Cholinergic receptors are divided into two major families: muscarinic receptors (mAChRs), which are G-protein-coupled, and nicotinic receptors (nAChRs), which are ligand-dependent ion channels. M1 mAChRs are the most abundant in the cortex and hippocampus and are involved in long-term potentiation (LTP), a neurobiological basis of learning. In AD, although M1 receptor levels may remain relatively stable, their coupling to second messengers is altered, suggesting post-receptor dysfunction. Thus, the M1 muscarinic acetylcholine receptor M1 shows reduced functional connectivity (FC) in the right posterior middle temporal gyrus in Aβ^+^ AD dementia patients compared to cognitively intact Aβ^−^ controls. On the other hand, nAChRs, especially the α4β2 and α7 subtypes, are significantly reduced in AD, contributing to synaptic dysfunction [35].

Cholinergic dysfunction not only affects cognition, but also influences neuroinflammation and amyloid homeostasis. Studies have shown that α7-nAChR receptor stimulation can reduce the release of proinflammatory cytokines by microglia, as well as promote Aβ uptake, acting as a clearance mechanism. However, the binding of Aβ to these same receptors can also induce neurotoxicity [35,109]. Similarly, soluble Aβ oligomers interfere with this pathway by enhancing the sequestration of estrogen receptors (ERα) in neurofibrillary tangles, a process that correlates with early p-tau217 elevation and exacerbates cholinergic depletion in the basal forebrain [78,79,110]

From a therapeutic perspective, the cholinergic hypothesis precipitated the development of acetylcholinesterase inhibitors (AChEIs), primarily donepezil, rivastigmine, and galantamine. Donepezil, approved for mild-to-severe AD, is typically administered at doses of 5 to 10 mg/day, with titrations up to 23 mg indicated for moderate-to-severe cases. Its primary adverse effects include nausea, diarrhea, insomnia, and bradycardia, as extensively documented in Phase III trials and comprehensive Cochrane reviews. Rivastigmine is indicated for mild-to-moderate AD and can be administered orally (6 to 12 mg/day) or via a transdermal delivery system (typically delivering 9.5 mg/24 h); the transdermal route is frequently preferred as it significantly mitigates gastrointestinal adverse effects and associated weight loss. Galantamine, also approved for mild-to-moderate stages at therapeutic doses of 16 to 24 mg/day, presents a side-effect profile comparable to that of donepezil. By inhibiting acetylcholinesterase, these pharmacological agents increase the synaptic availability of acetylcholine, thereby transiently ameliorating cognitive and behavioral deficits in patients with mild-to-moderate AD [111]. Furthermore, select AChEIs exhibit secondary molecular properties; notably, galantamine functions as a positive allosteric modulator of nicotinic acetylcholine receptors (nAChRs), a mechanism that potentiates its overall therapeutic efficacy [111]. Although their clinical utility remains symptomatic and they do not alter the underlying disease trajectory, AChEIs continue to be foundational to approved pharmacological regimens. Moreover, the concurrent administration of AChEIs with other targeted treatments, such as NMDA receptor antagonists, constitutes a standard polypharmacological strategy in the moderate-to-advanced stages of AD [112].

The use of selective M1 muscarinic receptor agonists has also been investigated, with the intention of improving cognitive function without producing the peripheral adverse effects associated with M2/M3 mAChRs [113,114]. Some compounds such as xanomeline, have shown some results [115].

### 3.2. Glutamatergic System

The glutamatergic system is the main excitatory system of the CNS and plays an essential role in synaptic plasticity, learning and memory [116]. In AD, alterations in glutamatergic neurotransmission have been linked to neurotoxicity, synaptic dysfunction and progression of neuronal degeneration. Glutamate acts on three main types of ionotropic receptors: NMDA (N-methyl-D-aspartate), AMPA (α-amino-3-hydroxy-5-methyl-4-isoxazolepropionic acid) and kainate, as well as on several metabotropic receptors (mGluR). NMDA receptors, in particular, are critical for LTP, a fundamental mechanism for memory consolidation [20,117].

Glutamate uptake is impaired in the vicinity of Aβ plaques in the cerebral cortex of APP/PS1 mice, leading to chronically elevated brain glutamate levels. This dysfunction in glutamate uptake may mediate aberrant neuronal activity in AD. In 5xFAD mice, significantly reduced amounts of glutamate have been observed in cerebral cortex tissue, indicating alterations in neurotransmitter homeostasis [116]. Recent studies confirm that glutamate recycling between neurons and astrocytes is essential for maintaining neurotransmitter homeostasis, and alterations in this homeostasis, resulting in excitotoxicity and neuronal death, have been described as a potential mechanism in the pathophysiology of AD [6,116,118]. Astrocytes play a key role in glutamate homeostasis, being responsible for 80–90% of synaptic glutamate uptake into the synaptic cleft [119]. In AD patients, glutamate transport activity was reduced in the cerebral cortex, and an association between reduced glutamate transport activity and increased Aβ accumulation was observed [37]. Circulating biomarker studies suggest that serum glutamate levels may correlate with mild cognitive impairment and AD. A study involving 783 participants [120] found that lower serum glutamate levels could predict progression from mild cognitive impairment to AD, although serum glutamate may not be an ideal peripheral biomarker.

#### 3.2.1. Excitotoxicity and NMDA Receptors

In AD there is an imbalance in glutamatergic signaling, characterized by a tonic overstimulation of NMDA receptors due to increased extracellular levels of glutamate, which triggers a phenomenon known as excitotoxicity. This excitotoxicity is primarily driven by Aβ oligomers, which inhibit the astrocytic glutamate transporter GLT-1. Rising plasma levels of p-tau181 and p-tau217 serve as markers for this phase, reflecting the synergistic impact of amyloid and tau on excitatory-inhibitory imbalance [78,110]. Additionally, this condition results in excessive calcium entry into neurons, activating signaling pathways leading to mitochondrial damage, oxidative stress and cell death [1,121]. In addition, synaptic and extrasynaptic distribution of NMDA receptors in postmortem brains has been described in AD patients. Compared with healthy individuals, people with AD have a lower number of NMDA receptors at synapses and increased numbers in extrasynaptic membranes [36].

**Figure 4:**
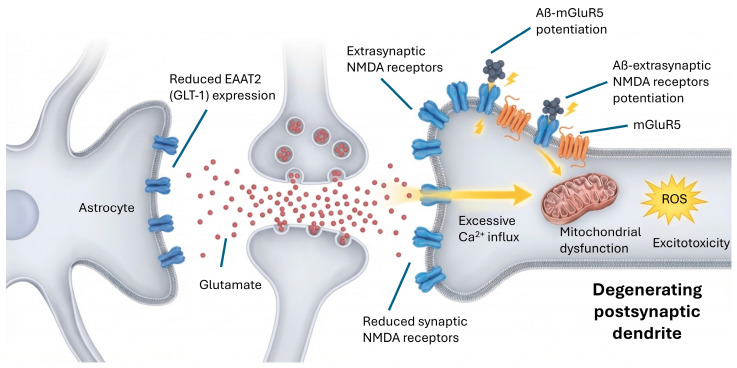
Astrocyte-mediated glutamate clearance is impaired through reduced expression/function of the high-capacity transporter EAAT2/GLT-1, elevating extracellular glutamate and increasing tonic NMDA receptor drive. In parallel, AD is associated with a maladaptive redistribution of NMDA receptors (reduced synaptic NMDARs and increased extrasynaptic NMDARs), biasing signaling toward Ca^2+^ overload, mitochondrial dysfunction, oxidative stress, and pro-death transcriptional processes rather than long-term potentiation. Furthermore, Aβ-driven potentiation of glutamatergic toxicity (including mGluR5-related mechanisms) integrates peptide pathology with excitatory synapse failure.

One of the causes of increased extracellular glutamate levels is the reduction in its uptake by astrocytes, due to decreased expression of the EAAT2 (GLT-1) transporter (Figure 4). Recent studies confirm that the WNT/β-catenin pathway is down-regulated in AD, resulting in increased oxidative stress and neuroinflammation, as well as decreased EAAT2 activity leading to neuronal death [122,123]. In addition, Aβ oligomers have been described to interact directly with NMDA receptors, potentiating their activity and exacerbating toxicity. A recent study demonstrated that Aβ oligomers modify the contribution of NR2B to NMDA receptor composition and function in early stages of AD through an integrin β1- and PKC-dependent pathway [38,124]. It has also been shown that Aβ can alter the distribution of AMPA receptors on the synaptic membrane, reducing synaptic efficacy and negatively affecting plasticity. These alterations precede synapse loss and contribute to the cognitive impairment characteristic of AD [125,126,127,128].

#### 3.2.2. Metabotropic Glutamate Receptors (mGluR5)

Metabotropic glutamate receptor 5 (mGluR5) localizes to excitatory synapses and glial cells. Binding of the tracer [18F] FPEB to mGluR5 has been found to be significantly lower in the hippocampus of AD patients compared to cognitively normal individuals [129]. This reduction in mGluR5 could be indicative of nonspecific synaptic loss. In addition, mGluR5 density showed a spatial association with tau pathology in AD [19]. Using the tracer [18F]PSS232, it was also shown that mGluR5 availability was significantly reduced in the hippocampus and parahippocampal gyrus of AD patients compared to normal controls [130]. The availability of mGluR5 correlated not only with neuropathological biomarkers of AD but also with neurodegenerative biomarkers and cognitive performance, suggesting that mGluR5 may be a novel neurodegenerative biomarker. Currently, abnormal mGluR5 signaling and associated synaptic failure are considered pathophysiological mechanisms of AD. Further studies show that Aβ42 oligomers interact with an mGluR5/cellular prion protein (PrPC) complex to disrupt physiological signal transduction of mGluR5, and mGluR5 appears to act as a co-receptor for AD-associated Aβ oligomers for both TBS-soluble extracts of AD brains and synthetic Aβ oligomers [131].

#### 3.2.3. Therapeutic Approaches

Memantine, a low-affinity, voltage-dependent uncompetitive NMDA receptor antagonist, is widely utilized for the management of moderate-to-severe stages of AD [1,121]. By preferentially blocking the pathological, prolonged activation of these receptors while sparing transient physiological signaling, memantine preserves synaptic function and mitigates glutamate-induced excitotoxicity [132]. The standard therapeutic dosage ranges from 10 to 20 mg per day, requiring a gradual titration from an initial dose of 5 mg to optimize tolerability. Commonly reported adverse effects include dizziness, headache, constipation, and hypertension. Furthermore, while not strictly contraindicated, its administration necessitates significant dose reductions and cautious clinical monitoring in patients presenting with severe renal impairment [133,134]. A meta-analysis of more than 24,000 patients showed that the use of memantine in patients with dementia may be associated with a reduction in all-cause mortality [135]. The combination of memantine with acetylcholinesterase inhibitors has shown additional benefits in some patients. Also, the combined use of donepezil and memantine significantly increases the likelihood of 5-year survival compared to no drug treatment or treatment with donepezil or memantine alone [133,134]. Research has focused on allosteric modulation of metabotropic glutamate receptors, with the goal of restoring excitatory balance without causing unwanted side effects. The therapeutic potential of mGluR5 as a target in AD has been widely recognized, and recent studies continue to validate the efficacy of various allosteric modulators of mGluR5 in ameliorating memory deficits and mitigating disease pathology [130,131,136].

Strategies to increase the expression or functionality of the EAAT2 transporter are also being investigated in preclinical models. One promising compound is sulbactam, which has been shown to protect neurons against the dual neurotoxicity of Aβ and glutamate loading by up-regulating GLT-1 expression [137]. Other strategies include compounds capable of modulating the intracellular signaling cascade associated with NMDA toxicity. Although these approaches have shown some potential, they have not yet been translated into effective clinical therapies [137].

### 3.3. GABAergic System

The GABAergic system is the major inhibitory system in the CNS and plays a key role in modulating neuronal activity, preventing hyperexcitability and maintaining synaptic homeostasis [116]. Its main neurotransmitter, gamma-aminobutyric acid (GABA), is synthesized from glutamate by the enzyme glutamate decarboxylase (GAD), and exerts its effects through two main types of receptors: GABAA (ionotropic) and GABAB (metabotropic).

For years, the GABAergic system has been considered relatively resistant to the neurodegenerative processes of AD compared to the cholinergic or glutamatergic systems. However, more recent studies have revealed significant alterations in GABAergic neurotransmission, especially in advanced stages of the disease. Already in 5xFAD mice, it was observed that GABA amounts were significantly reduced in the cerebral cortex, suggesting alterations in neurotransmitter homeostasis and a possible dysfunction in the glutamate/GABA-glutamine cycle [116]. In humans, deficit of GABAergic interneurons with AD has been shown to contribute to neuronal network dysfunction and cognitive impairment in AD [41,138]. In fact, the use of drugs affecting GABAA receptors is associated with the risk of developing AD and dementia, with a relative risk of 1.21 for AD and 1.15 for dementia [139]. In any case, it is important to assess alterations in GABA levels, as GABA accumulates rapidly during the post-mortem period [42,116,118,139].

#### 3.3.1. GABAergic Interneurons and Synaptic Dysfunction

Under normal conditions, GABAergic interneurons in the hippocampus and cerebral cortex play an essential role in regulating cortical rhythms and synchronizing neuronal activity, key processes for encoding memory and attention [140,141]. In AD, although the number of GABAergic neurons may be largely preserved, there are alterations in GAD expression, GABA availability and the functionality of its receptors [142].

**Figure 5:**
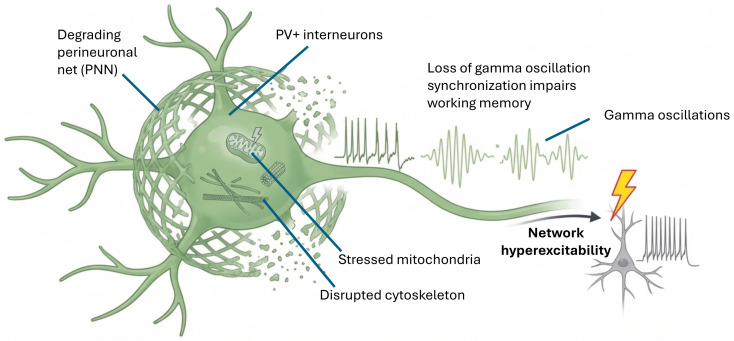
Parvalbumin fast-spiking interneurons (PV+) are vulnerable in AD, with several stressors including mitochondrial dysfunction, cytoskeletal disruption, and impaired signaling pathways that collectively compromise high-frequency inhibitory firing. Degradation or loss of perineuronal nets (PNNs), specialized extracellular matrix structures that stabilize synapses and protect PV interneurons from oxidative stress, further weakens inhibitory control, undermining gamma-band synchronization critical for working memory and cognitive integration. The resulting reduction in coordinated inhibition shifts cortical/hippocampal circuitry toward hyperexcitability and seizure susceptibility, linking microcircuit failure to network-level dysfunction.

In this regard, it appears that parvalbumin-expressing (PV+) interneurons are particularly vulnerable in AD, suffering alterations in mitochondrial metabolism, synaptic and cytoskeletal disruption, and decreased mTOR-mediated signaling [39]. These cells are crucial for generating gamma oscillations, which are involved in various cognitive functions. Dysfunction of PV+ interneurons can lead to hyperexcitability and increased susceptibility to seizures due to loss of synchronization of gamma oscillations in the hippocampus, a phenomenon linked to impaired working memory, and related to decreased GABAergic efficacy [40,143] (Figure 5). Indeed, gamma oscillations (25–100 Hz) have emerged as an early biomarker and potential therapeutic target in AD. Gamma entrainment through auditory and visual sensory stimulation can effectively attenuate AD pathology and improve cognitive function in murine models [144]. Similarly, targeted optogenetic stimulation of PV+ interneurons can restore gamma oscillations and memory function in AD models [145].

#### 3.3.2. GABAA Receptors and Amyloid Pathology

GABAA receptors, formed by pentamers of different subunits, mediate rapid inhibition through chloride influx and are affected by the presence of Aβ. This peptide can alter the expression and synaptic localization of these receptors, affecting tonic inhibition and causing an imbalance between excitation and inhibition that favors hyperexcitability and subclinical epileptic seizures observed in some AD patients [18,146]. Synaptic dysfunction in AD has also been linked to changes in GABA receptors, with a functional loss of GABAA receptors observed in the brains of AD patients [8]. GABAA-mediated slow synaptic inhibition is significantly reduced in granule neurons of the dentate gyrus of aged AD mice compared to wild-type controls and young AD mice, explaining the change in excitatory-inhibitory balance [147]. This alteration could be due to changes in receptor trafficking or plasma membrane damage induced by Aβ oligomers [148], but recently it has been proposed that the enzyme BACE1 (beta-site amyloid precursor protein cleaving enzyme 1) is responsible for GABAA receptor malfunction and neuronal hyperexcitability leading to loss of tonic GABAergic currents that alter the excitatory-inhibitory balance [149].

Modulation of GABAergic transmission may therefore also serve as a therapeutic pathway [1], as its alterations are clearly linked to synaptic dysfunction in this disease [1,8,18]. GABAA/BZ receptor density is also associated with regional susceptibility to tau accumulation [19].

#### 3.3.3. Perineuronal Networks (PNNs) and AD

An emerging area of research focuses on perineuronal networks (PNNs), extracellular matrix structures that encapsulate inhibitory cells and neurites in critical brain regions. PNNs have gained attention for their crucial role in synaptic stabilization and excitatory-inhibitory balance, and when disrupted, serve as a potential trigger for synaptic imbalance associated with AD [150]. It appears that the loss of PNNs is facilitated by microglia in the brains of AD models and humans [151]. AD-resilient individuals exhibit distinct changes in PNNs, which may contribute to preservation of cognition despite neuropathology [152]. PNNs especially protect PV+ interneurons from oxidative stress and maintain their rapid firing properties, being crucial for local GABAergic inhibition.

#### 3.3.4. Therapeutic Approaches

From a therapeutic point of view, benzodiazepines, which act as positive allosteric modulators of GABAA receptors, have traditionally been used in sleep and anxiety disorders in AD patients. However, their prolonged use has been associated with cognitive worsening, falls, and increased risk of dementia, limiting their clinical utility [153,154,155]. A meta-analysis confirmed that benzodiazepine use is associated with a slightly increased risk of dementia, particularly with long-term use, although high cumulative exposure did not appear to increase the risk of dementia in a dose-dependent manner [155]. Another longitudinal study found that cumulative exposure to benzodiazepines was minimally associated with increased risk of dementia, but did not increase in a dose-dependent manner [156].

The role of modulators of the GABAergic system, including selective partial agonists of specific GABAA receptor subunits (such as α2 and α3), which could improve cognitive function without the adverse effects of benzodiazepines, has also been investigated [157]. Positive allosteric modulation of α5-GABAA receptors seems to prevent age-related cognitive dysfunction [158].

Riluzole increases astrocytic glutamate transport activity and enhances glutamate clearance, which could indirectly benefit GABAergic function [159,160]. This compound has also been shown to restore GAD 65/67 (key enzyme in GABA synthesis) levels in 3xTg AD mice, bringing them closer to the levels of healthy controls [159,160,161]. In general, the experimental compounds seek to restore lost or desynchronized GABAergic inhibitory activity.

Targeted optogenetic stimulation of cortical GABAergic interneurons has also been shown to restore NREM sleep, reduce Aβ deposition, normalize neuronal calcium homeostasis, and improve memory function in murine models of AD [162]. It appears that microglia reprogramming occurs which ameliorates pathological phenotypes by increasing Aβ clearance by these cells. Optogenetics and PV+ interneuron modulators have also been used in murine models of AD to restore gamma rhythms and improve cognitive function, and optogenetic stimulation of PV+ interneurons during memory retrieval is sufficient to rescue memory deficits without the need to reduce plaque load, suggesting that memory dysfunction in AD is related to defects in retrieval rather than encoding [145,162].

More recently, the role of monoamine oxidase B (MAO-B) in reactive astrocytes has been explored, as elevated MAO-B activity enhances GABA production, leading to excessive tonic inhibition that disrupts neuronal excitability and synaptic function. MAO-B inhibition emerges as a targeted therapeutic approach to regulate astrocytic GABA synthesis and restore neural circuit homeostasis [163,164,165].

### 3.4. Serotonergic System

The serotonergic system, whose main neurotransmitter is serotonin (5-hydroxytryptamine or 5-HT), plays a crucial role in the regulation of mood, sleep, appetite, cognition and emotional behavior. Its neuronal bodies are mostly located in the raphe nuclei of the brainstem, from where they project widely to the cerebral cortex, hippocampus, basal ganglia and other CNS regions [46].

**Figure 6:**
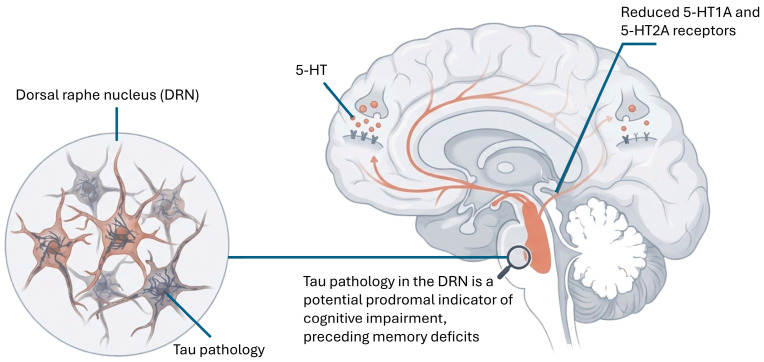
Dorsal raphe nucleus (DRN) serotonergic neuron degeneration and early tau involvement, positioning serotonergic disruption as a prodromal contributor to neuropsychiatric symptoms (depression, sleep disturbance, agitation) and subsequent cognitive decline. Reduced serotonergic receptor availability (including 5-HT1A/5-HT2A-related signaling changes) and diminished serotonergic release are shown to alter cortical/hippocampal plasticity, mood-regulating circuitry, and potentially Aβ/tau biology through downstream neuromodulatory pathways. Tau pathology in the DRN is positioned as a potential prodromal indicator of cognitive impairment, preceding memory deficits.

In AD, the serotonergic system undergoes early and progressive alterations leading to degeneration of serotonergic neurons in the dorsal raphe, as well as decreased serotonin levels in the hippocampus and prefrontal cortex, contributing to the neuropsychiatric symptoms of the disease, such as depression, agitation and sleep disorders [44]. In the hippocampus of 3xTg-AD mice, significant decreases in 5-HT levels were also observed [44,166]. In the hippocampus of postmortem brain samples from individuals with AD, dysfunction of the serotonergic system also appears. The dorsal raphe nucleus (DRN) is among the first regions to show tau pathology during AD progression, with 57% of control individuals and 90% of AD cases showing significant immunoreactivity for phosphorylated tau in this region [20,45]. Tau pathology in DRN may be a prodromal indicator of cognitive impairment in AD. When human P301L tau was overexpressed in the DRN of mice, depression-like behaviors and hyperactivity were observed without deficits in spatial memory, suggesting that behavioral changes may precede cognitive impairment in AD [45,46] (Figure 6).

#### 3.4.1. Serotonergic Receptors and Risk Factors

In addition to neuronal loss, alterations in the expression and function of serotonergic receptors occur. There are at least 14 subtypes of 5-HT receptors grouped into seven families, with both excitatory and inhibitory functions. In fact, variants in genes for serotonin receptors are considered possible risk factors for AD [22]. For example, a reduction in 5-HT1A receptor density in the hippocampus, a region involved in memory consolidation, has been described [167,168,169].

Serotonin improves biomarkers of neurogenesis, hippocampal volumes and cognitive functions in individuals with AD, with higher serotonin levels associated with higher brain and hippocampal volumes, which in turn correlated with better cognitive performance [170]. Initial positive associations were also found between serotonin and biomarkers related to neurogenesis and neuroplasticity, such as CNTF, FGF-4, BMP-6 and MMP-1, although these correlations lost statistical significance after adjustment for multiple comparisons [170].

Likewise, decreased 5-HT2A receptors in the prefrontal cortex are associated with dysfunctions in cognitive processing and affective disorders. PET neuroimaging studies have evidenced that 5-HT1b, 5-HT2a, 5-HT4 and 5-HT6 receptor density showed a strong spatial association with tau pathology, supporting the idea that alterations in the 5-HT system are central not only to cognitive impairment, but also to behavioral symptomatology. Significant reductions in 5-HT1B/1D and 5-HT6 receptor densities have been found in cortical areas of AD patients, where 5-HT1B/1D receptor density correlated with MMSE decline in frontal cortex [43,46,171]. Recent advances in the study of 5-HT2A receptors have revealed their potential role in psychedelic therapies for AD [172]. Research with psilocybin shows that this compound, acting as a 5-HT2A receptor agonist, promotes increased neuroplasticity, decreased inflammation, and improvements in cognitive functions such as creativity, cognitive flexibility, and facial emotional recognition [173]. Although 5-HT2A receptor density is reduced in AD, activation of these receptors by specific agonists has shown significant neuroprotective effects on hippocampal neurons through anti-apoptotic and anti-inflammatory pathways [173].

#### 3.4.2. Interactions with Amyloid and Tau Pathology

An important pathway of interaction between the serotonergic system and AD pathology is its relationship with Aβ deposits and tau protein. In animal models, stimulation of 5-HT4 and 5-HT6 receptors has shown beneficial effects on synaptic plasticity and reduced amyloidogenic processing of APP, thereby decreasing Aβ production [168]. More recent studies have shown that activation of 5-HT4 receptors by agonists such as prucalopride and RS-67333 significantly reduces tauopathy, decreases synaptic tau, increases proteasome activity, and improves cognitive functioning in PS19 mice (tauopathy model). Activation of 5-HT4R stimulates adenylate cyclase to produce cAMP, which then activates PKA, known to enhance CREB-mediated transcription and hyperphosphorylate 26S proteasomes, conferring enhanced degradation capacity for aggregation-prone proteins such as tau [169,174].

Altered expression of 5-HT3 receptors (HTR3A, HTR3C, HTR3E) could affect neurotransmission, synaptic plasticity and neuroinflammation, influencing cognitive outcomes. Escitalopram, a highly selective serotonin reuptake inhibitors (SSRIs), significantly reduced Aβ levels in mice by increasing α-secretase cleavage of APP. Chronic treatment with escitalopram significantly reduced plaque burden by 28% and 34% at doses of 2.5 and 5 mg/day, respectively, and completely arrested individual plaque growth over time [175].

Environmental enrichment (EE) in 5XFAD mice also increased 5-HT concentrations [105]. It appears that EE restores cognitive impairment by ameliorating AD pathology and up-regulating synapse-related proteins and neurotransmitters, including increases in acetylcholine and serotonin concentrations in the frontal cortex and hippocampus. However, it is important to note that some studies have shown that environmental enrichment may have paradoxical effects, exacerbating amyloid plaque formation in certain transgenic models of AD, possibly due to increased synaptic activity that increases Aβ production [105,176].

From a therapeutic point of view, SSRIs, such as sertraline or fluoxetine, have been used to treat depressive symptoms in patients with AD. Beyond their effect on mood, some studies suggest that SSRIs may have neuroprotective effects, possibly by modulating inflammation, promoting neurogenesis, or altering Aβ metabolism [175]. Prolonged SSRI use is associated with reduced plasma phosphorylated tau-181 levels and restoration of DRN metabolic activity in AD patients [177]. However, clinical results have been mixed. A meta-analysis demonstrated that SSRI-related antidepressants have significant advantages in the treatment of AD compared with placebo on scales such as NPI, CSDD and BPRS, although the MMSE showed no notable changes [175,177,178]. Trials such as ADMET (AD Antidepressant Treatment Trial) showed no significant differences between SSRIs and placebo in terms of mood improvement or cognitive progression [179]. On the other hand, it has also been proposed that antidepressants may accelerate cognitive decline in people with dementia, although some drugs appear to be less harmful than others. A large Swedish cohort study involving 18,740 patients with dementia found that current use of antidepressants was associated with faster cognitive decline, with SSRIs being the most commonly prescribed (64.8% of all antidepressant prescriptions). The study showed that escitalopram was associated with the fastest cognitive decline (−0.76 points/year on MMSE), followed by citalopram (−0.41 points/year) and sertraline (−0.25 points/year). In addition, higher doses of SSRIs were associated with increased risk of severe dementia, fractures, and all-cause mortality [178]. These contradictory findings underscore the need for additional research to clarify the effects of SSRIs on AD progression [180].

Despite this, serotonergic modulation continues to be an active area of research, particularly with the development of partial agonists and selective antagonists of specific receptors, such as 5-HT6, whose inhibition has been linked to improved cognitive performance in preclinical models [181]. Over the past two decades, the 5-HT6 receptor has received increasing attention and has become a promising target for improving cognition. Several studies with structurally different compounds have shown that both 5-HT6 receptor antagonists and agonists improve learning and memory in animal models [182]. It is increasingly clear that blockade of 5-HT6 receptors leads to improved cognitive performance in a wide variety of learning and memory paradigms, and also results in anxiolytic and antidepressant-like activity. These actions are primarily supported by improvements in cholinergic, glutamatergic, noradrenergic, and dopaminergic neurotransmission, along with learning-associated neuronal remodeling [178,183,184].

Several 5-HT6 antagonists have advanced to clinical development, and to date, at least three of them have reached phase II/III trials as drug candidates for cognitive enhancement. A preliminary clinical study reported that the cognitive enhancement properties of a 5-HT6 receptor antagonist (SB-742457) extend to patients with AD [185]. More recent advances include the development of selenium-containing compounds as 5-HT6 receptor antagonists, which have shown neuroprotective effects superior to those of approved donepezil, with antioxidant and glutathione peroxidase-like activity, in addition to regulating antioxidant and proinflammatory genes [184].

Serotonin and its receptors are thus shown to be a potential therapeutic strategy for neurodegenerative diseases [186]. Studies on the 5-HT7 receptor, the most recent receptor identified in the serotonergic receptor family, suggest its potential as a therapeutic target with neuroprotective effects by modulating signaling pathways related to excitotoxicity, oxidative stress, apoptosis and synaptic plasticity [186,187,188].

Regarding its utility as a biomarker, serotonin has a crucial role in modulating brain structure and cognitive functions in individuals with AD; higher serotonin levels are associated with preserved whole-brain and hippocampal volumes, which in turn correlate with better cognitive performance [170]. Furthermore, positron emission tomography (PET) neuroimaging studies have demonstrated that the loss of serotonergic terminals correlates directly with the severity of neuropsychiatric symptoms in AD, reinforcing the premise that alterations in the 5-HT system are integral not only to cognitive impairment but also to behavioral symptomatology. Consequently, molecular imaging of serotonergic targets emerges as a viable modality for the early detection of AD and the assessment of disease progression [129]. Notably, the cortical density of 5-HT1B, 5-HT2A, 5-HT4, and 5-HT6 receptors exhibits a strong spatial correlation with the distribution of tau pathology [19].

Complementing these pharmacological insights, advancements in structural biology and nanoscale imaging are facilitating the high-resolution elucidation of the molecular constituents involved in AD pathogenesis. Cryo-electron microscopy (cryo-EM) has recently resolved the high-resolution structures of amyloid fibrils extracted from human brain tissue, revealing distinct polymorphs that may correlate with specific clinical phenotypes [189]. These structural determinations hold significant implications for understanding how Aβ and tau interact with neurotransmitter receptors at the synaptic level. Additionally, emerging nanoscale techniques, such as atomic force microscopy (AFM)-based nanomechanical mapping, permit the visualization of Aβ aggregation dynamics and their subsequent impact on neuronal membrane biophysics at subcellular resolution [190]. Such methodological approaches may ultimately enable the early detection of pathological protein conformations prior to the onset of overt clinical symptoms, while concurrently serving as robust platforms for screening compounds designed to modulate neurotransmitter receptor–amyloid interactions.

It is pertinent to acknowledge that the physiological functions of serotonin extend beyond the central nervous system (CNS). In the periphery, serotonin is a critical regulator of cardiovascular function, gastrointestinal motility, and platelet aggregation. The dysregulation of peripheral serotonergic systems is implicated in diverse pathologies, including pulmonary arterial hypertension (where elevated serotonin drives vascular remodeling), carcinoid syndrome, and irritable bowel syndrome. This systemic dimension is highly relevant to AD, as peripheral serotonin disturbances may modulate CNS function via gut–brain axis signaling, immune system regulation, and cerebrovascular effects, mechanisms that fundamentally intersect with AD pathophysiology. Nevertheless, a comprehensive analysis of peripheral serotonin biology remains beyond the scope of the present review.

### 3.5. Dopaminergic System

The dopaminergic system plays a crucial role in the regulation of motivation, learning, motor control, reward and certain aspects of executive functioning [172]. Although it has traditionally been associated with diseases such as Parkinson’s or schizophrenia, it has also been implicated in the pathophysiology of AD, especially with regard to apathy, executive dysfunction and other neuropsychiatric symptoms [172,191]. Alterations in dopamine-related FC may be associated with worse cognitive performance [35,49,192,193,194,195].

Dopaminergic neurons are mainly located in the midbrain, in nuclei such as the substantia nigra and ventral tegmental area, from where they project to cortical and subcortical structures, including the striatum, prefrontal cortex and hippocampus. There are five subtypes of dopaminergic receptors, classified into two families: D1-like (D1 and D5) and D2-like (D2, D3 and D4), all of which are coupled to G proteins. Recent studies have shown that dopaminergic receptor expression shows age- and brain region-specific changes, with D1 receptors showing a more pronounced decrease with age compared to D2 receptors. The relative availability of D1/D2 receptors in associative cortexes is negatively correlated with age and positively correlated with spatial working memory performance [49,192,193,194,196].

**Figure 7:**
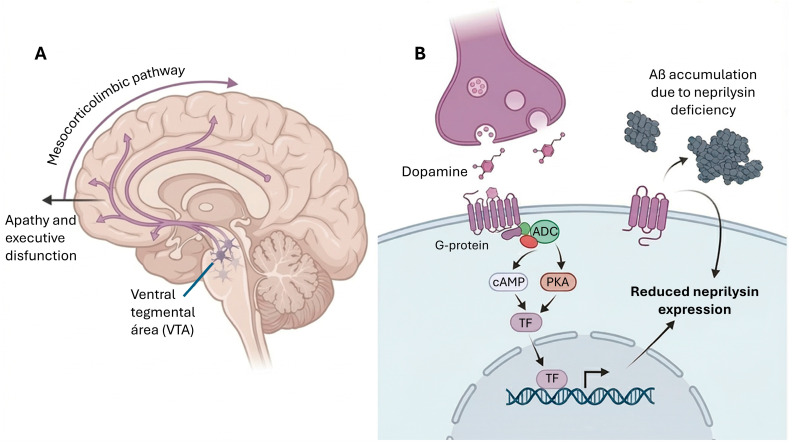
(**A**) Degeneration and hypofunction of ventral tegmental area (VTA) dopaminergic neurons within the mesocorticolimbic pathway reduce dopaminergic modulation of prefrontal and limbic circuits that support motivation, reward processing, and executive control, thereby contributing to apathy and dysexecutive phenotypes in AD. (**B**) Dopamine-dependent regulation of amyloid catabolism via neprilysin-dopaminergic signaling increases neprilysin abundance/activity in a brain-region-specific manner, promoting enzymatic degradation and promoting brain region-specific neprilysin-dependent Aβ degradation; dopaminergic failure is therefore positioned as a pathogenic amplifier of Aβ accumulation. This model integrates neurotransmission, proteostasis, and peptide clearance to explain why early monoaminergic degeneration may influence both neuropsychiatric symptoms and core proteinopathies.

In AD, a significant loss of dopaminergic neurons has been documented in the ventral tegmental area, a region whose degeneration has been correlated with dopamine depletion in the hippocampus and prefrontal cortex, contributing to deficits in memory and motivation. This loss is early and precedes the onset of clinical symptoms, suggesting that the dopaminergic system may be involved in the early stages of cognitive impairment [195]. Early and progressive dysfunctions of the ventral tegmental area (VTA) dopaminergic system have been described in AD, particularly during the long presymptomatic phase [50,195]. Degeneration of VTA dopaminergic neurons, due to reduced hippocampal dopaminergic innervation, impairs parvalbumin interneuron (PV-IN) firing and gamma waves, weakens inhibition of pyramidal neurons, and induces hippocampal hyperexcitability through reduced D2 receptor-mediated activation of the CREB pathway [50]. VTA dopaminergic neurons show hyperexcitability in 3xTg-AD mice due to up-regulation of casein kinase 2 (CK2), which alters ion channels and contributes to behavioral abnormalities in AD [47,50] (Figure 7).

In the mesocorticolimbic pathway (MCL), the AD dementia group showed lower dopamine D1 receptor-related FC in the left precuneus than the AD mild cognitive impairment (MCI) group [35]. Regression models indicated a positive association between episodic memory scores and dopamine D2 receptor-related FC in the retrosplenial and temporo-occipital regions. The observed dopaminergic dysfunction between the MCL system and the posterior default mode network (DMN) could be mediated by dopamine D1 receptor distribution. Dopamine D1 receptor density was also associated with regional susceptibility to tau accumulation [35]. Connectivity alterations in functional acetylcholine and dopamine pathways develop in parallel with cognitive decline in AD and could be a clinically relevant marker in early AD [35].

Reduced dopamine levels, as well as alterations in dopamine receptor signaling, have been associated with impaired performance on tasks requiring cognitive flexibility, working memory, and decision making. In animal models, hippocampal dopaminergic dysfunction has been directly linked to impaired spatial memory [197,198].

From a clinical perspective, one of the most prevalent and limiting symptoms in AD is apathy, which has been associated with dysfunction of the mesocorticolimbic dopaminergic pathway. Apathy is manifested by reduced motivation, initiative, and emotional responsiveness, and is associated with worse functional and cognitive outcome [23,197,198].

Neuroimaging studies in AD patients have shown a decrease in vesicular dopamine transport (VMAT2) and D2 receptor in cortical regions, reinforcing the hypothesis of a functional impairment of this pathway [199]. Recent longitudinal studies have shown that the magnitude of striatal D2 receptor decline was approximately 50% of previous cross-sectional estimates (~4% vs. ~8% per decade), suggesting that the rate of D2 receptor decline has been overestimated in previous cross-sectional studies. Furthermore, it has been suggested that the interaction of Aβ with dopaminergic neurons could induce oxidative stress and mitochondrial dysfunction, accelerating neuronal degeneration [193]. Using SPECT with 123I-FP-CIT, apathy in AD was also shown to correlate with the dopamine transporter. Significant inverse correlations were observed between binding potential values and apathy scores in the left caudate nucleus, suggesting that the pathological basis of apathy could be impairment of the dopaminergic nervous system. Functional connectivity biomarkers have revealed that D2 receptor losses correlated across selected striatal and extrastriatal regions and were associated with changes in cerebrovascular parameters, observed mainly in regions particularly susceptible to vascular insults, such as the basal ganglia and hippocampus [193,200].

#### 3.5.1. Therapeutic Approaches

Dopaminergic therapy in AD is still limited. Some studies have explored the use of dopaminergic agonists, such as rotigotine, showing modest improvements in apathy and behavioral symptoms in patients with mild to moderate AD [201,202,203]. A pilot study in 30 patients with mild AD and 10 healthy controls evaluated the effects of rotigotine (4 mg/day for 4 weeks) and rivastigmine on cortical plasticity using transcranial stimulation [204]. Rotigotine normalized reduced levels of LTP-type cortical plasticity (LTP) in AD patients, effects that were not observed with rivastigmine or placebo. In 7 patients retested after 12 weeks of rotigotine treatment, there were improvements in cognitive and executive function [201,203]. A phase 3 clinical trial involving 348 patients with mild to moderate AD is currently ongoing in Italy, evaluating the combination of transdermal rotigotine (4 mg/day) with rivastigmine. This prospective, randomized, double-blind, placebo-controlled 24-week study evaluates global cognition as the primary outcome, with results expected by 2025–2026. However, described side effects are application site reactions, nausea, hypotension and hallucinations (risk in elderly). Phase II showed improvement in cortical plasticity [202]. A meta-analysis showed that rotigotine, piribedil, and pramipexole have been reported to improve apathy levels in Parkinson’s disease, and similar studies are being explored in AD [201].

##### MAO-B Inhibitors

On the other hand, compounds such as selegiline, a monoamine oxidase B inhibitor (MAO-B inhibitor), have shown in some studies the ability to improve behavioral symptoms and slow deterioration in early stages of the disease, probably by elevating synaptic levels of dopamine and reducing oxidative stress [163,205,206,207,208,209]. Selegiline confirmed protective effects against anxiety and Aβ-induced cognitive deficits. Selegiline (0.5 mg/kg/day for 30 days) improved memory performance, reduced anxiety, and modulated oxidative-antioxidant status in AD rats [210]. The findings suggest that selegiline may alleviate anxiety-like behavior and Aβ-induced cognitive deficits through modulation of oxidative-antioxidant status. Its safety has also been evaluated [211]. Described side effects include insomnia, agitation and cardiovascular alterations.

The development of novel tacrine-selegiline hybrids with cholinesterases (ChEs) and monoamine oxidases (MAOs) inhibitory activities as multifunctional AD drugs have also shown improved properties. Compound 7d exhibited balanced activity against ChEs and MAOs, excellent blood–brain barrier permeability, and improved cognitive function of mice with scopolamine-induced memory impairment [48,97].

However, important limitations of irreversible MAO-B inhibitors such as selegiline in long-term treatment have also been identified. Prolonged treatment with selegiline fails to significantly reverse spatial memory deficits due to compensatory mechanisms involving increased levels of the enzyme D-amino acid oxidase (DAO), which reverses the beneficial effects of tonic GABA. In contrast, reversible MAO-B inhibitors such as KDS2010 maintain their long-term efficacy without activating these compensatory mechanisms [207].

##### Dopamine and Amyloid Degradation

It has been suggested that indirect modulation of the dopaminergic system through physical exercise or transcranial direct current stimulation (tDCS) may have beneficial effects on executive function and mood in AD, possibly by increasing endogenous dopamine release [204,212]. The dopaminergic system promotes neprilysin-mediated degradation of Aβ in the brain. Dopamine and the dopamine precursor levodopa (L-DOPA) induced Aβ degradation in the brain by increasing the amount and activity of neprilysin [213]. Chemogenetic activation of VTA neurons increased neprilysin abundance and activity and reduced Aβ deposits in the prefrontal cortex in a neprilysin-dependent manner. In aged mice, they were found to have less dopamine and neprilysin in the anterior cortex, a decrease that was accentuated in AD model mice. Treatment of AD model mice with levodopa reduced Aβ deposition and improved cognitive function. These observations demonstrate that dopamine promotes brain region-specific neprilysin-dependent Aβ degradation, suggesting that dopamine-associated strategies have the potential to address this aspect of AD pathology [213].

##### Physical Exercise and Dopamine

Accumulating evidence indicates that exercise can prevent multiple pathological features found in AD and improve cognitive function through delaying the degeneration of cholinergic and monoaminergic neurons; increasing levels of acetylcholine, norepinephrine, serotonin, and dopamine; and modulating the activity of certain neurotransmitter-related G-protein-coupled receptors [212]. Intense exercise for 6 months has been shown to induce significant brain changes in patients with early and mild Parkinson’s disease. A consistent increase in available dopamine transporter (DAT) sites in the substantia nigra was observed in 90% of participants, along with an increase in neuromelanin signal in the substantia nigra pars compacta, indicating neuromodulatory effects of exercise on the dopaminergic system [214,215,216,217,218,219,220].

Animal studies have shown that voluntary endurance exercise (wheel running) for 5 months leads to a severe reduction in Aβ deposition and tau phosphorylation in the hippocampus of AD mice. This process is associated with a significant decrease in APP phosphorylation and presenilin 1 expression. Exercise reduces AD-type neurodegeneration by inactivating the glycogen synthase kinase 3 (GSK3) signaling pathway [212]. Aerobic exercise has been shown to increase hippocampal volumes by 1–2% and improve executive function scores by 5–10% in older adults, while resistance training improves cognitive control and memory performance by 12–18% in older individuals [214,217,218]. Walking for 8 weeks may have positive effects on some neurotransmitters, including dopamine, and reduce the level of depression in elderly women with AD [221].

##### Transcranial Stimulation

Bifrontal transcranial direct current stimulation (tDCS) induces neurotransmitter release in polysynaptically connected subcortical areas [222]. A study confirmed that cognitive enhancement under the tDCS condition is related to endogenous dopamine release, providing clinico-biological evidence demonstrating that enhancement of dopaminergic signaling by tDCS in the ventral striatum is associated with improvement in executive function [223]. Active tDCS (20 min, 2 mA) induced a significant decrease in [11C]raclopride binding in the striatum compared to sham tDCS, suggesting an increase in extracellular dopamine in a part of the striatum involved in the reward-motivation network [101,224]. Repetitive prefrontal tDCS may activate dopaminergic neurons in the VTA, leading to increased hippocampal dopamine release and increased DAT levels in the nucleus accumbens. Importantly, prefrontal tDCS completely restored long-term potentiation (LTP) deficits in the hippocampal CA3-CA1 pathway in Tg2576 mice, as well as cognitive and noncognitive deficits, including object recognition memory [101]. Another study demonstrated that tDCS has substantial efficacy in improving general cognition in patients with Parkinson’s disease, particularly in executive function and language [224,225].

Animal studies have shown that cathodal, but not anodal, tDCS for 10 min increased extracellular dopamine levels for more than 400 min in the striatum of anesthetized rats, suggesting that tDCS has a direct and/or indirect effect on the dopaminergic system in the rat basal ganglia [101,226].

### 3.6. Noradrenergic System

The noradrenergic system, whose main neurotransmission is mediated by noradrenaline (NA), plays key roles in the regulation of attention, alertness, sleep-wakefulness, emotional modulation and stress response. In the CNS (CNS), most noradrenergic neurons are located in the locus coeruleus (LC), a brainstem structure that projects widely to the cerebral cortex, hippocampus, cerebellum and spinal cord [51]. In the hippocampus of 3xTg-AD mice, significant decreases in norepinephrine (NE) levels were observed [7]. ADRA2A and ADRA1D genes are associated with norepinephrine and epinephrine signaling mechanisms [22,227].

In AD, the noradrenergic system undergoes significant early degeneration. Dysregulation of noradrenergic signaling has been implicated in impaired cognitive processes [22]. The LC is, in fact, one of the first regions to accumulate deposits of hyperphosphorylated tau protein, even before overt clinical signs of cognitive impairment appear [52]. Loss of noradrenergic neurons in this region has been correlated with the severity of dementia and with the presence of neuropsychiatric symptoms such as agitation, anxiety and sleep disturbances [228]. The LC appears to be the most vulnerable structure in the CNS to aging-related factors, leading to early LC death and cognitive impairments [228]. Understanding the action of noradrenaline in brain cells, particularly in astrocytes that exhibit a high density of adrenergic receptors, is a future strategy to develop new drugs to mitigate neurodegeneration and cognitive decline [227].

Changes in LC integrity precede tau accumulation in the medial temporal lobe, and together these processes are associated with lower cognitive performance. LC integrity and tau accumulation in the hippocampus predicted cognitive changes approximately 3 years later [228,229]. The study demonstrated that spatiotemporal patterns of LC integrity predict cortical tau deposition following the progression described in Braak’s staging [228]. The LC is one of the earliest sites of hyperphosphorylated tau accumulation prior to the allocortical regions in AD, with evidence that changes in LC integrity precede tau accumulation in the medial temporal lobe. The specific pattern of tau spreading from the LC to medial temporal areas is associated with common gene expression profiles that map biological functions in the regulation of protein transport [228].

Loss of neuromelanin has also been described in the earliest stages of AD, even before the neuroinflammatory reaction [227,230]. Changes in LC with increasing Braak stage included increased neuronal and microglial Iba1 loss along with a reduction in neuromelanin, dopamine β-hydroxylase (DβH) and tyrosine hydroxylase (TH). Interestingly, in LC, increased hyperphosphorylated tau and loss of neuromelanin were detected from Braak stage III-IV. Indeed, the use of neuromelanin-MRI has been proposed as a sensitive technique to identify early changes in AD and even as a promising biomarker of catecholaminergic function, offering a non-invasive approach to visualize and quantify the structural and functional integrity of the LC and thereby aiding in the diagnosis and quantification of longitudinal disease changes. It could also provide a stratification tool to predict the treatment success of pharmacological interventions targeting the dopaminergic and noradrenergic systems [230,231].

#### 3.6.1. Function of Noradrenergic Receptors

Noradrenaline acts on α- and β-adrenergic receptors, both presynaptic and postsynaptic, which are distributed in multiple brain regions. Stimulation of these receptors influences synaptic plasticity, memory consolidation and neurogenesis. In animal models, noradrenergic stimulation improves spatial memory and learning, whereas its depletion induces cognitive deficits [54,232]. Alterations in monoaminergic systems with AD thus participate in neuropsychiatric symptoms. Disruptions in functional connectivity, particularly within the default mode network, are associated with alterations in monoaminergic signaling involving norepinephrine, and these disruptions contribute to symptoms such as depression, anxiety, and behavioral changes observed in AD patients [54]. Patients with early-onset AD (EOAD) show higher NPI scores, lower LC integrity, and similar CSF norepinephrine levels compared to late-onset AD (LOAD). Notably, EOAD exhibited lower LC integrity regardless of disease stage, and LC integrity correlated negatively with neuropsychiatric symptoms. Noradrenaline levels were increased in AD correlating with AD biomarkers [233].

**Figure 8:**
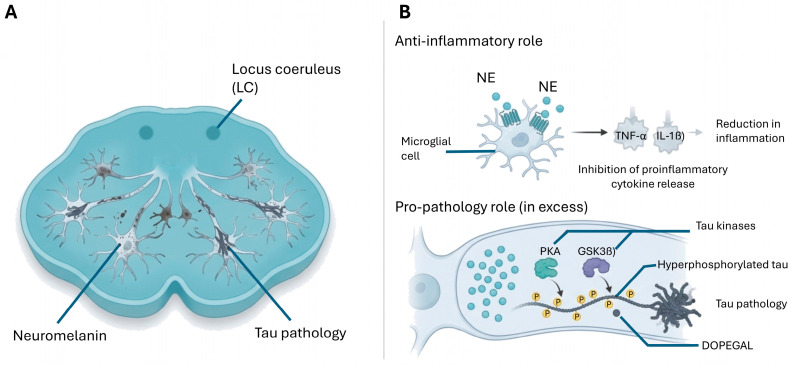
(**A**) Progressive degeneration of noradrenergic neurons that project from locus coeruleus (LC) to cortex/hippocampus and regulate attention, arousal, and stress responses, due to tau pathology. (**B**) Physiological norepinephrine (NE) can suppress microglial pro-inflammatory cytokine release and constrain neuroinflammation, whereas excessive/prolonged NE exposure may promote tau pathology via kinase activation (e.g., PKA, GSK3β) and toxic catsecholaldehyde metabolism (e.g., DOPEGAL), thereby accelerating neurodegeneration. Early LC pathology can simultaneously remove an anti-inflammatory neuromodulator and create conditions that facilitate tau propagation.

Loss of noradrenergic tone in AD also contributes to increased neuroinflammation. Noradrenaline exerts an anti-inflammatory effect by modulating microglial activation and suppressing the production of proinflammatory cytokines. Dysfunction of this system promotes chronic microglial activation, sustained release of interleukins and TNF-α, and progressive neuronal damage [54] (Figure 8).

#### 3.6.2. Therapeutic Approaches

Therapeutically, several studies have explored the utility of potentiating the noradrenergic system in AD. One approach has been the use of selective noradrenaline reuptake inhibitors (SNRIs), such as atomoxetine, which have shown improvements in executive functions and attention in patients with mild to moderate AD, although results have been variable [54,232,234,235,236,237,238].

A phase II clinical trial used atomoxetine (40–80 mg/day) as a potential disease-modifying therapy in subjects with mild cognitive impairment due to AD [234]. The results showed that atomoxetine was safe, well tolerated, and achieved target engagement in prodromal AD. Thus, atomoxetine was associated with a significant 5–6% reduction in CSF levels of total tau and pTau181 compared to placebo, providing biomarker evidence for potential slowing of neurodegeneration. CSF levels of panels of brain biomarkers of synaptic function, brain metabolism and glial immunity also showed effects with treatment. At the level of brain metabolism, increases in FDG-PET were observed in key medial temporal lobe circuits [234]. The study determined plasma atomoxetine levels by mass spectrometry at concentrations within therapeutic ranges, with median plasma atomoxetine concentrations of 224.4 and 313.8 ng/mL during the active treatment phase. Treatment with atomoxetine resulted in expected and marked increases in CSF levels of norepinephrine and dopamine, the primary substrates for the NE transporter [234].

Additional studies have shown that atomoxetine produces dual effects on midbrain glial cells, inducing a tonic inhibition of microglial activation and proinflammatory gene expression, while concurrently promoting the synthesis of astrocyte neurotrophic factors, including significant increases in GDNF and BDNF. However, it has also been shown that high concentrations of atomoxetine can induce oxidative stress and alter mitochondrial function in differentiated neuronal cells [54,236]. Furthermore, side effects included increased blood pressure/heart rate, dry mouth and insomnia.

The use of β-adrenergic agonists and α2 antagonists, such as yohimbine, has also been studied to increase NA release and enhance cognition, although their clinical use is limited by adverse effects such as hypertension or anxiety. More recently, LC stimulation by neuromodulation techniques (such as vagal stimulation or transcranial stimulation) has been investigated, with promising results in preclinical models [208,229,239,240,241,242]. Even, neurobiology studies have shown that LC characteristics are linked to response to VNS in drug-resistant epilepsy. In patients with better response to therapy, trends toward lower activity and higher contrast were found in specific portions of the LC. Greater integrity of LC-hippocampal connections was found in patients with better response to treatment [208,241]. On the other hand, transcutaneous auricular vagus nerve stimulation (taVNS) induces LC responses measured by fMRI that are coupled to changes in salivary alpha amylase, a marker of noradrenergic activity [241,243]. Additional studies have shown that 4 s of taVNS trains reliably induce increased pupillary dilation, an indication of increased LC-norepinephrine system activity [241]. The underlying mechanisms of VNS include activation of the nucleus of the solitary tract (NTS) and consequently of noradrenergic neurons in the LC, resulting in the release of NE in brain structures involved in memory formation such as the hippocampus, basolateral amygdala, and medial prefrontal cortex. Studies have shown that VNS improves cognitive function by suppressing inflammatory responses and activating the cholinergic anti-inflammatory pathway, resulting in the inhibition of proinflammatory cytokines such as TNF-α and IL-6 [208,239,240,241,242].

#### 3.6.3. Paradoxical Effects of Excessive Norepinephrine and LC Vulnerability

LC degeneration has also been linked to increased vulnerability to other neurodegenerative processes and reduced cognitive reserve capacity. Prolonged exposure to excessive norepinephrine in the brain has been shown to induce tau aggregation, neuronal death, and cognitive deficits in early tau transgenic mice [244]. The study administered reboxetine (RBX), a norepinephrine reuptake inhibitor, to increase NE levels in early tau transgenic (ADLPTau) mice for two months. Only RBX-treated mice exhibited cognitive deficits, and immunohistochemical analysis revealed increased aggregates of hyperphosphorylated tau in the LC and hippocampus. Western blot analysis showed that RBX injections led to overactivation of tau kinases PKA and GSK3β, resulting in hyperphosphorylated tau, neuronal loss, and cognitive impairments [245]. Thus, excessive NE exposure accelerates tau pathology through overactivation of tau kinases [244]. Additional studies with human brain organoids exposed to higher concentrations of NE also showed elevated hyperphosphorylated tau and increased activity of the same tau kinases, confirming that the findings in mice translate to human systems [244,246].

Specific molecular mechanisms underlying the selective vulnerability of LC to tau pathology have been identified. 3,4-dihydroxyphenylglycolaldehyde (DOPEGAL), a metabolite of NE produced exclusively in noradrenergic neurons by monoamine oxidase A (MAO-A) metabolism, activates asparagine endopeptidase (AEP) that cleaves tau at residue N368 into forms prone to aggregation and spreading. DOPEGAL triggers aggregation of tau cleaved by AEP in vitro and in intact cells, resulting in LC neurotoxicity and spread of pathology to the forebrain [247]. DOPEGAL is up-regulated in the LC of human AD brains and is toxic to noradrenergic neurons. Similarly, prevention of DOPEGAL production by siRNA silencing of DBH attenuated AEP activation and tau-induced cell death [247].

#### 3.6.4. Regulators of Neurotransmitter Release

Novel regulators of neurotransmitter release, the intracellular and non-GPI-anchored isoforms of neuronal CD59, termed IRIS-1 and IRIS-2 (Isoforms Rescuing Insulin Secretion), have recently been identified [7,227,248]. They were found to be present in neurons and astrocytes in the human brain [7,187]. Silencing of IRIS-1 and 2 in SH-SY5Y cells reduces the formation of the SNARE complex, essential for synaptic vesicle exocytosis, which decreases noradrenaline secretion [7,187]. Decreased expression of neuronal IRIS-1 and 2 has been observed in AD patients and in non-demented individuals with type 2 diabetes. In addition, silencing of all CD59 isoforms (including IRIS-1 and 2) elevates phosphorylated tau and expression of cyclin-dependent kinase 5 (CDK5), a key promoter of tau hyperphosphorylation and accumulation in AD [7,187]. Prolonged exposure to high concentrations of glucose or cytokines markedly reduces IRIS-1 and 2 expression in SH-SY5Y cells, suggesting a link between AD pathology and metabolic stress through modulation of these isoforms. A link is thus established between altered metabolism, neurotransmitter function and tau pathology in AD, suggesting that IRIS isoforms could be potential therapeutic targets to preserve noradrenergic function and prevent AD progression [7,187].

### 3.7. Histaminergic System

The histaminergic system, less well understood than other neurotransmitter systems, plays a modulatory role in multiple functions of the CNS, including sleep and wakefulness regulation, appetite control, cognition, thermoregulation, and stress response [249]. Histaminergic neurons are located exclusively in the posterior mammillary tubercle of the hypothalamus, specifically in the tuberomammillary nucleus, from where they diffusely project to the cerebral cortex, hippocampus, thalamus, basal ganglia and brainstem.

**Figure 9:**
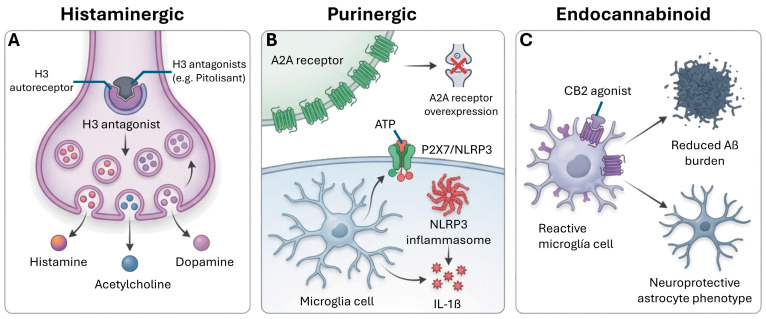
(**A**) Histaminergic modulation via H3 autoreceptors, where H3 antagonism (e.g., pitolisant-like mechanisms) can enhance release of histamine and other transmitters (ACh, dopamine, NE), thereby boosting arousal and cognitive processing. (**B**) Purinergic signaling highlighting A2A receptor overexpression-linked synaptic impairment and microglial P2X7/NLRP3 inflammasome activation by pathological ATP elevations, driving IL-1β-mediated neuroinflammation. (**C**) Endocannabinoid control of glial reactivity via CB2 receptor signaling in microglia, promoting reduced Aβ burden and shifting astrocytes toward neuroprotective phenotypes, consistent with an anti-inflammatory, homeostatic role for endocannabinoid pathways.

Histamine exerts its action through four receptor subtypes (H1–H4), all coupled to G proteins. In the CNS, H1 and H3 receptors are the most relevant: H1 has excitatory postsynaptic effects, while H3 acts as an inhibitory autoreceptor that regulates the release of histamine and other neurotransmitters such as acetylcholine, dopamine and noradrenaline [55]. Detailed description of histaminergic neurons in the tuberomammillary nucleus has confirmed that they provide histamine to the whole brain via extensive fiber projections. Activation of histaminergic neurons of the TMN activates glutamatergic cells of the subiculum that project to the retrosplenial granular cortex mainly through the histamine H2 receptor, participating in alertness-driven accelerated locomotion [57] (Figure 9A).

In AD, a significant reduction in histamine levels in the brain has been observed, as well as degeneration of wakefulness-promoting neurons, including histaminergic neurons located in the tuberomammillary nucleus of the posterior hypothalamus. Thus, sleep fragmentation with shortening of N3 sleep appears along with excessive daytime sleepiness and twilight syndrome [56,250], with this neuronal loss contributing to the sleep–wake cycle dysfunction, daytime fatigue and cognitive impairment observed in these patients. Histamine depletion may also be involved in impaired appetite and body weight control, frequent symptoms in advanced AD [250].

Therapeutic interventions targeting the histaminergic system have shown potential to slow and correct neurodegenerative processes in AD. Melatonin up-regulation, known to be effective in improving cognitive function and preventing Aβ accumulation, together with the involvement of the histaminergic system in cognition and memory, becomes notable for promoting neurotransmission in AD [20].

#### 3.7.1. Histamine Receptor Antagonists

There is evidence that the H3 receptor is overexpressed in certain brain regions in AD models, which could contribute to decreased histaminergic tone and reduced release of other key neurotransmitters for cognition [251]. For this reason, H3 receptor antagonists have been proposed as potential procognitive agents. By blocking this receptor, the release of histamine and other excitatory neurotransmitters is enhanced, improving synaptic transmission and neuronal plasticity. Several preclinical studies have shown that H3 antagonists, such as cyproxyphan or pitolysant, can improve memory and attention in animal models of dementia [248]. These effects have been attributed to both increased histamine and secondary enhancement of acetylcholine and dopamine levels in the prefrontal cortex and hippocampus.

Pitolisant, an H3 receptor antagonist/inverse agonist clinically approved for narcolepsy (18–36 mg/day), reverses AD-like pathophysiology and ameliorates cognitive deficits in a murine model of AD. Behavioral assays and in vivo wide-field Ca^2+^ imaging revealed that recognition memory, learning flexibility, and slow-wave impairment improved after 15-day treatment with pitolisant [23,252]. Improved recognition memory correlated closely with slow-wave coherence, suggesting that slow waves serve as a biomarker for treatment response and AD drug screening [23,252]. Furthermore, pitolysant reduced Aβ deposition and dystrophic neurites around plaques, and enhanced neuronal lysosomal activity, whose inhibition blocked cognitive and slow-wave restoration. It was previously confirmed that pitolysant has the potential to treat various neuropsychiatric disorders by increasing histamine release in most brain regions, including the cerebral cortex. H3 receptor antagonists/inverse agonists enhance memory consolidation and retrieval, as well as restore the retrieval of forgotten memories long after learning and forgetting [253]. Described side effects are insomnia, anxiety and gastrointestinal alterations.

Although there are no approved histamine-based treatments for AD yet, clinical trials have been conducted with the H3R antagonists ABT-288, GSK239512 and MK0249 in different populations with mild cognitive impairment or mild AD [254]. More recently, new pitolysant-derived sulfonyl compounds have been developed for AD, confirming that only five currently approved pharmacological treatment options have been shown in clinical studies to improve cognitive function in AD patients [255].

H1 receptors may also be involved in the neuropsychiatric symptomatology of AD. Some first-generation H1 antihistamines, such as diphenhydramine, cross the blood–brain barrier and may worsen cognitive impairment [256]. Indeed, their use is associated with an increased risk of developing dementia in patients aged ≥ 65 years, with an adjusted hazard ratio of 1.782 (95% CI = 1.368–2.168, *p* < 0.001) [257]. Mild non-amnestic cognitive deficits are consistently induced by first-generation antihistamines and tricyclic antidepressants, whereas benzodiazepines cause combined amnestic and non-amnestic deficits [153,257,258].

With respect to second-generation antihistamines, fexofenadine has a lower incidence of CNS-level side effects, including sedation. Other compounds, such as loratadine, did not alter cognitive function at recommended doses but did at doses higher than recommended [259,260] (Figure 9A).

#### 3.7.2. Histamine and Neuroinflammation

There is evidence that histamine may act as a modulator of the immune system in the CNS and thus of neuroinflammation. Through H1 and H4 receptors, histamine can influence microglial activation and proinflammatory cytokine release, although the exact effects seem to depend on the context and differential expression of these receptors [254]. Indeed, there is a dual role for histamine in modulating microglial inflammatory responses. Histamine per se triggers microglial motility, while it prevents LPS-induced microglial migration and IL-1β release. This assigns a new putative anti-inflammatory role for histamine, acting via H4R to restrain exaggerated microglial responses under an inflammatory state [261]. Another study confirmed that histamine regulates microglia in vivo, via the H4 receptor. Hdc knockout mice chronically deficient in histamine have microglia with reduced branching in the striatum and reduced expression of the H4 receptor. IGF-1 expressing microglia are particularly reduced; however, the microglial response to LPS stimulus is enhanced in Hdc knockout mice [262].

Also histamine stimulates human microglia to alter cytokine release, specifically by increasing levels of IL-8 and IL-6, two key inflammatory mediators involved in neuroinflammation and microglial inflammatory signaling [263].

### 3.8. Purinergic System

The purinergic system includes a complex network of signals mediated by purines such as adenosine triphosphate (ATP) and adenosine, which act as neurotransmitters and neuromodulators in the CNS (CNS). This system regulates processes such as neurotransmission, synaptic plasticity, neurogenesis, inflammatory response and cell death [58,59,264].

Purinergic receptors are divided into two major families: P1 receptors, which respond to adenosine, and P2 receptors, which respond to ATP and other nucleotides. P1 receptors include A1, A2A, A2B, and A3, while P2 receptors are subdivided into P2X (ion channels) and P2Y (G-protein-coupled receptors) [58,59,264].

In AD, the purinergic system is involved in multiple aspects of pathogenesis, particularly neuroinflammation, excitotoxicity and synaptic modulation. Adenosine, through the A2A receptor, has an important effect on the release of neurotransmitters such as glutamate and dopamine, and on synaptic plasticity in the hippocampus, a key region for memory [60].

A2A receptor overexpression has been observed in the hippocampus/entorhinal cortex rather than in the frontal cortex in AD patients, which is not observed in age-matched control brains, indicating an association with AD pathology [60,62]. This overactivation contributes to synaptic impairment, dysregulation of glutamatergic transmission and potentiation of Aβ-induced neurotoxicity. In animal models, A2A blockade improves memory and reduces neuroinflammation [62].

Early neuronal up-regulation of A2AR in the presence of ongoing amyloid pathology exacerbates memory deficits in APP/PS1 mice. These behavioral alterations were not related to major changes in the development of amyloid pathology, but rather associated with increased phosphorylated tau in neuritic plaques [265]. Proteomic and transcriptomic analyses indicated that neuronal up-regulation of the receptor promoted neuronal and non-neuronal autonomic alterations, including enhanced neuroinflammatory response but also loss of excitatory synapses and impaired neuronal mitochondrial function [60,265] (Figure 9B).

#### 3.8.1. P2X7 Receptors and Neuroinflammation

P2X7 receptors, activated by high extracellular concentrations of ATP, play a crucial role in the activation of microglia and the release of proinflammatory cytokines such as IL-1β and TNF-α. In AD, increased P2X7 expression has been demonstrated in affected brain areas such as the hippocampus and entorhinal cortex [266]. Sustained activation of the P2X7 receptor can exacerbate chronic inflammation and accelerate neuronal death. The P2X7 receptor in microglia has been described to mediate neuroinflammation through NFκB activation and NLRP3 inflammasome-dependent regulation of inflammasome-dependent inflammation [267]. The up-regulated P2X7 receptor of microglia participates in mediating and regulating neuroinflammation, with activation of P2X7R triggering downstream mechanisms only when extracellular ATP reached pathological concentrations [268]. These changes are completely inhibited by nimodipine, a potent blocker of cellular damage caused by monomeric and oligomeric Aβ [267]. Taurodeoxycholate (TDCA), a GPCR19 ligand, inhibited the priming phase of N3I activation, suppressed P2X7R expression and P2X7R-mediated Ca^2+^ mobilization and N3I oligomerization, essential for IL-1β/IL-18 production by microglia [61].

More recently, however, it has been proposed that the P2X7 receptor drives microglial activation and proliferation. Contrary to previous reports describing P2X7R as a “death receptor,” the study provided evidence for a novel trophic role for the P2X7R pore in microglia [268,269]. In any case, antagonism of this receptor has been proposed as a therapeutic strategy in AD. Preclinical trials have shown that the use of P2X7 antagonists reduces Aβ load, microglial reactivity and improves performance in cognitive tasks [61,266,267,268,269,270].

#### 3.8.2. A1 Receptor and Neuroprotection

A1 receptor, which generally has neuroprotective effects, appears decreased in AD, which could contribute to down-regulation of neuronal excitability and increased excitotoxicity. A1 activation reduces glutamate release and stabilizes synaptic homeostasis, but its functional impairment favors Aβ-induced damage and oxidative stress [271,272]. Adenosine A1 and A2A receptors are expressed in the human brain and have a proposed involvement in the pathogenesis of dementia. Their preclinical targeting may mitigate Aβ and tau neurotoxicity while improving cognition and memory [273].

#### 3.8.3. Therapeutic Approaches

Therapeutically, one of the most investigated compounds in relation to the purinergic system is caffeine, a non-selective antagonist of A1 and A2A receptors. Several epidemiological studies have indicated that moderate caffeine consumption is associated with a lower risk of developing AD, and preclinical studies have shown improvements in cognition after chronic administration [274].

Caffeine prevents adenosine receptors from being activated by regulating brain functions such as sleep, cognition, learning and memory. Experimental work performed in vivo and in vitro provides evidence that caffeine exerts its neuroprotective effects through antagonistic binding to A2A receptors (A2ARs) [275].

In transgenic mice models of AD, caffeine, after 4 months of administration, ameliorates memory deficits and pathology by reducing hippocampal Aβ content through inhibition of β- and γ-secretase expression and generating Aβ monomers, protecting against Aβ-induced neurotoxicity [276]. This protective effect was also confirmed in “aged” APPsw mice already showing signs of mental decline, after 4–5 weeks of caffeine intake [275,276].

Other drugs in development focus on specific A2A and P2X7 inhibitors. Istradefylline, a selective A2A antagonist approved for Parkinson’s disease (20–40 mg/day), has demonstrated procognitive effects in animal models of AD. Prefrontal cortex dopamine levels and cognitive performance were significantly reduced by 6-OHDA injury. Istradefylline, donepezil and methamphetamine improved cognitive performance of rats with prefrontal cortex lesion by increased prefrontal cortex dopamine levels in both normal and lesioned rats [277].

Recently, attempts have been made to translate the cognitive enhancement of A2AR antagonists in rodents and non-human primates, and the safety profile of A2AR in phase IIB-III clinical trials to reevaluate and repropose the ability of istradefylline to control cognitive impairment in traumatic brain injury and Parkinson’s disease [22,278]. Described side effects are dyskinesia, dizziness and hallucinations.

### 3.9. Endocannabinoid System

The endocannabinoid system is a neuromodulatory signaling network widely distributed in the CNS, with key functions in the regulation of synaptic homeostasis, neuronal plasticity, neurogenesis, stress response, inflammation and pain control [279,280].

This system is composed of endocannabinoids, mainly anandamide (AEA) and 2-arachidonylglycerol (2-AG), synthesized “on demand” from membrane lipids; cannabinoid receptors, such as CB1 (major in CNS neurons) and CB2 (mainly in glial and immune cells); degradative enzymes such as fatty acid amide hydrolase (FAAH) and monoacylglycerol lipase (MAGL), which regulate the duration of the cannabinoid signal [19]. The CB1 receptor, highly expressed in glutamatergic and GABAergic synapses of the hippocampus and cortex, regulates neurotransmitter release. Its moderate stimulation improves synaptic plasticity, although chronic activation can lead to cognitive impairment [281]. In addition, it modulates oxidative stress and promotes hippocampal neurogenesis.

It has been described that AD patients present decreased expression levels of CB1 receptor and increased levels of 2-AG and its degradative enzyme MAGL compared to healthy controls. Subgroup analysis revealed significantly lower levels of CB1R in AD, particularly in studies using western blotting and in studies evaluating CB1R in the frontal cortex [15]. In 3xTgg-AD mice, altered CB1 levels appear to be age and/or pathology dependent. At 6 and 12 months of age, CB1 mRNA levels were significantly higher in the prefrontal cortex, dorsal hippocampus and basolateral amygdaloid complex, but lower in the ventral hippocampus [63].

The CB2 receptor appears overexpressed in reactive microglia of affected regions, suggesting a role in neuroinflammatory modulation [64,282]. CB2 activation in animal models reduces microglial activation, proinflammatory cytokine production and Aβ plaque burden [283]. Microglial stimulation of the CB2 receptor improves cognitive dysfunction in App^NL-G-F/NL-G-F^ mice by controlling astrocytic activation and inducing beneficial neuroinflammation. Chronic treatment with JWH 133, a selective CB2 agonist, for 12 weeks significantly improved cognitive performance and reduced Aβ deposition [284]. Transcriptomic analysis showed that CB2 stimulation suppressed the expression of A1 neurotoxicity-related genes in astrocytes and promoted the expression of A2 neuroprotection-related genes [284].

Down-regulation in retrograde endocannabinoid signaling pathways has also been observed for certain receptor genes (ADRA1D, HTR3A, HTR3C and HTR3E). This suggests a possible involvement or alteration in this type of signaling in AD [22]. However, in a model that investigated whether the pattern of receptor densities in different neurotransmitter systems, including CB1, was related to tau-PET Z-score in Aβ-positive participants, linear regression analysis did not reveal a statistically significant association between higher tau-PET Z-score and lower CB1 receptor density in the models evaluated [19] (Figure 9C).

#### Therapeutic Strategies

Increasing endocannabinoid levels by FAAH or MAGL inhibition shows cognitive benefits in preclinical models [285]. FAAH and MAGL inhibitors alone or in combination appear to produce neuroprotection by reversing cognitive deficits along with Aβ-induced neuroinflammatory, oxidative responses and neuronal death, delaying AD progression [286,287]. In murine models of APP/PS1 and Tg2576 amyloidosis, enhancement of AEA signaling by genetic deletion of FAAH delayed cognitive deficits in APP/PS1 mice and improved cognitive symptoms in 12-month-old AD-like mice. Chronic pharmacological inhibition of FAAH completely reversed neurocognitive decline, attenuated neuroinflammation, and promoted neuroprotective mechanisms in Tg2576 mice [65].

With respect to MAGL inhibition, systemic administration of JZL184, a potent irreversible MAGL inhibitor, resulted in 6- to 7-fold increases in 2-AG levels in the brain, and the production and accumulation of total Aβ and Aβ42 peptides and the expression of BACE1, a key enzyme of Aβ synthesis, were significantly reduced in 5xFAD mice [288].

Using positron emission tomography (PET), age-, sex-, and pathology-related dynamic changes in the availability of CB1 and MAGL from early stages of Aβ pathology were observed. Findings showed that [18F]FMPEP-d2 (CB1) availability was significantly reduced in AppNL-G-F mice compared to wild type at 12 months (late stage pathology), suggesting that the endocannabinoid system is a useful target for AD diagnostics and treatment [289].

Clinically, cannabidiol (CBD) has shown neuroprotective and anti-inflammatory effects without psychoactive properties [290]. CBD can reduce Aβ accumulation and tau hyperphosphorylation, suggesting the possibility of delaying AD progression. CBD provides neuroprotective effects through cannabinoid and non-cannabinoid receptors [291]. In addition, CBD demonstrates significant effects in promoting neuroplasticity, particularly in brain regions such as the hippocampus [292,293]. CBD showed ability to rescue the Wnt/β-catenin pathway, linked to tau hyperphosphorylation, and reduce iNOS protein synthesis and NO release. In addition, the ability of CBD to reduce reactive gliosis may be associated with its role as an inverse agonist at the CB2 receptor [294].

Preliminary studies with THC and CBD have reported mild improvements in noncognitive symptoms such as agitation and insomnia [295]. Evaluation of the efficacy and safety of low-dose oral THC for the treatment of neuropsychiatric symptoms related to dementia was evaluated in patients receiving THC 4.5 mg daily and placebo for 3 weeks. The results showed no significant difference between THC and placebo in symptom reduction, although THC was safe and well tolerated [296,297].

The combination of THC and CBD has shown promising results in improving memory and reducing plaque deposition in animal models. However, most studies have limitations, such as small sample sizes, lack of standardization in dosages, and lack of double-blind randomized clinical trials [298].

The potential of cannabis-based treatment to improve the quality of care for palliative care-eligible patients with dementia is currently being investigated [299]. The study is focusing on an oral drug called T2:C100, containing THC and CBD, to determine whether it can more effectively reduce agitation than a placebo (available in https://clinicaltrials.gov/study/NCT05644262?id=NCT05644262&rank=1 (accessed on 28 February 2026)).

## 4. Conclusions

The current understanding of AD has evolved beyond the classic Aβ and tau pathologies. It is now recognized as a complex, multifactorial condition, where a variety of biological processes, including glia-mediated neuroinflammation, cerebrovascular dysfunction, metabolic alterations, and significant synaptic loss, contribute to its pathogenesis. These changes may initiate decades before clinical symptoms manifest.

A central finding is that the malfunction of multiple neurotransmitter systems is a core component of AD, influencing both cognitive and neuropsychiatric symptoms. While the cholinergic system was the first to be linked to AD and led to initial treatments, its limited efficacy suggests that the disease is not adequately explained or managed by addressing a single system. Similarly, glutamatergic excitotoxicity and alterations in the GABAergic system contribute to neural network hyperexcitability and cognitive impairment. Beyond the most extensively studied networks, research has highlighted the early dysfunction and critical role of other neurotransmitter systems. For example, the degeneration of the dopaminergic and serotonergic pathways contributes to symptoms such as apathy and other neuropsychiatric manifestations, while also influencing Aβ and tau pathology. Notably, dopamine has been shown to promote Aβ clearance, whereas an excess of noradrenaline, originating from a system vulnerable in early disease stages, could accelerate tau pathology. Less explored systems, such as the histaminergic and purinergic networks, also emerge as promising therapeutic targets. H3 antagonists, such as pitolisant, have demonstrated the capacity to improve cognition and attenuate pathology in AD models. Likewise, the blockade of A2A receptors (e.g., via caffeine or istradephylline) could reduce neuroinflammation and enhance cognitive function. The endocannabinoid system, exhibiting protective and anti-inflammatory properties (e.g., cannabidiol), provides another avenue of investigative interest. Crucially, these neurotransmitter systems interact in a complex, bidirectional manner, dictating that dysfunction in one can exacerbate deficits in another. This interconnectedness elucidates why current monotherapies yield limited effects on disease progression; thus, the future of AD treatment necessitates multimodal approaches and precision medicine. This paradigm requires the utilization of advanced biomarkers for accurate patient stratification to tailor both pharmacological and non-pharmacological interventions, alongside the development of combination therapies targeting multiple pathological cascades simultaneously.

Despite the diversity of primary targets among the pharmacological agents utilized in AD (acetylcholinesterase inhibitors, NMDA antagonists, SSRIs, SNRIs, H3 antagonists, etc.), it is plausible that they share common downstream effector pathways. Two mechanisms are highlighted as points of convergence: neuroinflammation and the homeostasis of the excitatory/inhibitory (E/I) balance. Glial activity is modulated by multiple systems, for instance, noradrenaline derived from the locus coeruleus exerts a tonic anti-inflammatory effect, CB2 receptor activation in microglia reduces the expression of proinflammatory cytokines, and dopamine promotes Aβ degradation via neprilysin; thus, interventions targeting any of these pathways may attenuate the chronic neuroinflammatory state. Concurrently, the regulation of the E/I balance, which is altered in AD, may be improved by strategies that potentiate GABAergic inhibition, mitigate glutamatergic excitotoxicity, or enhance monoaminergic modulation. Consequently, the partial symptomatic efficacy of drugs with distinct primary mechanisms could be explained by their capacity to ultimately influence these shared final processes.

Beyond conventional pharmacology, innovative therapeutic approaches directly addressing network dysfunction are emerging. Sensory stimulation at 40 Hz (gamma) has demonstrated the capacity to recruit parvalbumin-positive interneurons, restore gamma oscillations, and diminish amyloid pathology in animal models, with clinical trials currently underway. This non-invasive approach acts directly upon the physiological substrate of neuronal desynchronization. Furthermore, multimodal strategies utilizing low-dose pharmacological combinations, or the development of multi-target directed ligands (MTDLs), could simultaneously address several impaired systems while minimizing adverse effects. In this context, albeit with requisite caution regarding vulnerable populations, compounds with potent monoaminergic profiles (such as specific psychedelics) are being investigated for their capacity to promote neuroplasticity and reduce neuroinflammation, thereby establishing a promising yet nascent investigative pathway.

The conceptualization of AD as a systemic failure dictates that its management cannot be strictly limited to pharmacological interventions. Environmental and social factors, including a Westernized diet, chronic stress, and social isolation, interact with biological vulnerabilities via neuroendocrine and epigenetic mechanisms to generate a cellular environment conducive to pathogenesis. Systemic inflammation and the microbiota–gut–brain axis are profoundly influenced by dietary patterns; sustained stress elevates cortisol levels and disrupts noradrenergic and serotonergic neurotransmission, while involuntary isolation deprives the cerebral cortex of essential synaptic stimulation. In animal models, environmental enrichment has been shown to up-regulate acetylcholine and serotonin concentrations and attenuate neuropathology. Therefore, a comprehensive multimodal approach should encompass psychosocial interventions, physical activity, and social support. The hypothesis that an absence of existential purpose can manifest as biological deterioration is a proposition that warrants rigorous interdisciplinary investigation, particularly to elucidate its molecular correlates.

Despite recent scientific advancements, fundamental inquiries remain unresolved. Is sufficient attention being directed toward the role of astrocytes, whose dysfunction, induced by metabolic stress, the accumulation of misfolded proteins, or impaired glutamate transport, could constitute an early catalyst for neurodegeneration? What specific role does the impairment of the glymphatic system, responsible for the clearance of cerebral metabolites during sleep, play in the accumulation of Aβ and tau proteins? How does cerebral energetic hypometabolism (detectable via FDG-PET) correlate with the inability of neurons to sustain neurotransmission? These unresolved questions suggest that, in its etiology, AD might primarily be a disorder of metabolic waste clearance and cellular bioenergetics rather than strictly a consequence of toxic protein overproduction. The forthcoming frontier will necessitate the integration of cellular biology, bioenergetics, and network dynamics to identify the fundamental systemic vulnerabilities of the pathology.

Moving forward, several concrete investigative and therapeutic trajectories emerge from this synthesis. Primarily, the development of multi-target directed ligands (MTDLs) that simultaneously engage multiple neurotransmitter systems, for instance, combining cholinesterase inhibition with 5-HT6 antagonism or H3 receptor modulation, represents a rational evolution from empirical polypharmacy to designed polypharmacology. This pharmacological advancement must be coupled with the integration of high-resolution neuroimaging biomarkers, such as receptor-specific PET tracers and neuromelanin-MRI for assessing locus coeruleus integrity, into clinical trial designs. Such integration is essential to enable accurate patient stratification based on predominant network dysfunction, thereby driving the transition toward precision medicine. In parallel, non-invasive neuromodulatory techniques, including gamma-frequency sensory stimulation, transcranial direct current stimulation (tDCS), and vagus nerve stimulation, warrant investigation as disease-modifying strategies targeting physiological desynchronization. Furthermore, the gut–brain axis constitutes a critical adjunctive frontier; because the intestinal microbiome modulates systemic inflammation and neurotransmitter bioavailability, targeted interventions utilizing prebiotics, probiotics, and dietary optimization offer significant therapeutic potential. These biological strategies should synergize with comprehensive, lifestyle-based multimodal interventions encompassing physical exercise, cognitive training, and social engagement, all of which necessitate validation through robust clinical trials. Ultimately, the overarching priority remains the implementation of these strategies during the preclinical stages of AD. Intervening during this therapeutic window, when neurotransmitter dysfunction may still be reversible and prior to irreversible synaptic attrition, requires the continuous refinement of fluid and molecular biomarkers to accurately identify vulnerable cohorts and strictly monitor target engagement in prevention protocols.

Finally, the profound correlation between neurotransmitter networks and the emotional and cognitive dimensions of human experience underscores that Alzheimer’s disease is not solely a biological process but also a complex phenomenon where molecular dysfunction directly intersects with our understanding of identity, cognition, and the sociological positioning of older populations.

## Data Availability

No new data were created or analyzed in this study. Data sharing is not applicable to this article.

## Abbreviations

The following abbreviations are used in this manuscript:

2-AG	2-Arachidonylglycerol
5-HT	5-Hydroxytryptamine
ACh	Acetylcholine
AChE	Acetylcholinesterase
AD	Alzheimer’s Disease
ADRA1A	Adrenergic receptor 1A
ADRA1D	Adrenergic receptor 1D
AEA	Anandamide
AEP	Asparagine Endopeptidase
AMPA	A-Amino-3-Hydroxy-5-Methyl-4-Isoxazolepropionic Acid
ATP	Adenosine Triphosphate
Aβ	Β-Amyloid
BDNF	Brain-Derived Neurotrophic Factor
CB1	Cannabinoid Receptor Type 1
CB2	Cannabinoid Receptor Type 2
CBD	Cannabidiol
ChAT	Choline Acetyltransferase
CNS	Central Nervous System
CSF	Cerebrospinal Fluid
DAT	Dopamine Transporter
DMN	Default Mode Network
DOPEGAL	3,4-Dihydroxyphenylglycolaldehyde
DRN	Dorsal Raphe Nucleus
DβH	Dopamine Β-Hydroxylase
EAAT2	Excitatory Amino Acid Transporter 2
EOAD	Early Onset AD
FAAH	Fatty Acid Amide Hydrolase
FC	Functional Connectivity
FDG	Fluorodeoxyglucose
GABA	Gamma-Aminobutyric Acid
GAD	Glutamate Decarboxylase
GDNF	Glial Cell Line-Derived Neurotrophic Factor
GSK3	Glycogen Synthase Kinase 3
IRIS-1	Isoforms Rescuing Insulin Secretion 1
IRIS-2	Isoforms Rescuing Insulin Secretion 2
LC	Locus Coeruleus
L-DOPA	Levodopa
LOAD	Late-Onset AD
LTP	Long-Term Potentiation
mAChRs	Muscarinic Receptors
MAGL	Monoacylglycerol Lipase
MAO-A	Monoamine Oxidase A
MAO-B	Monoamine Oxidase B
MCI	Mild Cognitive Impairment
MCL	Mesocorticolimbic Pathway
mGluR	Metabotropic Receptors
NA	Noradrenaline
nAChRs	Nicotinic Receptors
NE	Norepinephrine
NMDA	N-Methyl-D-Aspartate
NTS	Nucleus Of The Solitary Tract
PNNs	Perineuronal Networks
PV+	Parvalbumin-Expressing
ROS	Reactive Oxygen Species
SNRIs	Selective Noradrenaline Reuptake Inhibitors
SSRIs	Selective Serotonin Reuptake Inhibitors
taVNS	Transcutaneous Auricular Vagus Nerve Stimulation
tDCS	Transcranial Direct Current Stimulation
TH	Tyrosine Hydroxylase
TMN	Tuberomammillary Nucleus
VAChT	Vesicular Ach Transporter
VMAT2	Vesicular Dopamine Transport
VNS	Vagus Nerve Stimulation
VTA	Ventral Tegmental Area
